# Design and Evaluation of Sustainable Structural Lightweight Concrete Using Recycled Pet as Aggregates

**DOI:** 10.12688/f1000research.168557.2

**Published:** 2026-02-14

**Authors:** Chikadibia Kalu Awa Uche, Sani Aliyu Abubakar, Stephen Ndubuisi Nnamchi, Valentine Hyginus Udoka Eze

**Affiliations:** 1 Civil Engineering, Kampala International University - Western Campus, Bushenyi, Western Region, Uganda; 2 Mechanical Engineering, Kampala International University - Western Campus, Bushenyi, Western Region, Uganda; 3 Electrical, Telecommunications and Computer Engineering, Kampala International University - Western Campus, Bushenyi, Western Region, Uganda

**Keywords:** PET aggregates, mix design, structural lightweight concrete, sustainable construction

## Abstract

**Background:**

The accumulation of polyethylene terephthalate (PET) plastic waste poses environmental and sustainability challenges due to its non-biodegradable nature and limited disposal options. Repurposing PET as aggregates in structural lightweight concrete (SLWC) offers a dual benefit of waste valorisation and conservation of natural resources, while supporting circular economy objectives.

**Methods:**

Eight experimental mix proportions of SLWC were developed using a factorial design approach, with water–to–cement ratios ranging from 0.40 to 0.45. PET aggregates were produced via thermal–mechanical processing and subjected to calcium hypochlorite treatment to improve surface bonding with cement paste. Standard tests were conducted to determine workability (Vebe time), fresh and dry densities, compressive strength, splitting tensile strength, and water absorption. Structural efficiency was also computed as a strength-to-weight performance indicator.

**Results:**

The fresh and dry densities of the PET-based SLWC ranged from 1455–1515 kg/m ^3^ and 1490–1537 kg/m ^3^, respectively, corresponding to category D1.6 lightweight concrete. Compressive strengths ranged between 14.1 and 16.5 MPa, fulfilling the LC13 classification for structural applications. Splitting tensile strengths were between 0.84 and 1.4 MPa, with several mixes achieving minimum thresholds for structural performance. Water absorption values ranged from 4.66% to 10.16%, remaining within international standards for lightweight concrete durability. Workability was low (Vebe times 13–40 s), attributed to the angular and hydrophobic properties of PET aggregates. Structural efficiency values of 9.5–10.9 kPa·m ^3^/kg exceeded minimum international requirements.

**Conclusions:**

This study confirms that PET aggregates can be successfully utilised to produce structurally viable and durable lightweight concrete. Although workability is reduced, the compressive strength, tensile strength, density, and durability criteria align with international standards. These results demonstrate a sustainable strategy for PET waste valorisation in construction, contributing to resource conservation, reduced environmental burden, and advancement of circular economy goals.

## 1. Introduction

Waste directly results from human activity ( C. Uche et al., 2022). The challenge of waste disposal is the direct result of an increase in waste generation owing to technological advancements in modern lifestyles ( Gupta et al., 2014). Plastics are used in virtually all aspects of life and for many different purposes, including electrical appliances, car parts, construction materials, storage, and packaging ( Aocharoen & Chotickai, 2023). The global production of plastics reached an estimated 400 million metric tons in 2021, with polyethylene terephthalate (PET) representing a significant portion of plastic waste owing to its extensive production and widespread application in plastic bottles and food packaging ( Alves, 2023; Bachtiar et al., 2020). Approximately 500 million plastic bottles are discarded annually, which translates to approximately one million bottles per minute globally. This rate is projected to increase by 23% by 2025 ( Qaidi & Al-kamaki, 2020). Environmental pollution poses an aesthetic nuisance and can lead to the obstruction of drains and streams, the release of unpleasant odours from stagnant liquids in plastic containers, and the creation of breeding grounds for insects and rodents ( C. Uche et al., 2022). The large amount of plastic waste produced by human activities and its slow rate of degradation result in the demand for large expanses of land for landfills. Incineration is not a viable method of disposal because incomplete incineration of plastics releases dioxins (toxic fumes) into the air, which is detrimental to human health and the environment ( Bachtiar et al., 2020; Lee et al., 2019). Researchers are obliged to provide efficient, secure, cost-effective, and sustainable methods to address the significant increase in single-use plastic waste (PW) ( C. Zhang et al., 2023).

Recycling plastic waste and integrating it into construction materials is a viable solution for mitigating the challenge of plastic waste disposal ( Ragaert et al., 2017). Consequently, numerous researchers have examined the potential of using plastic waste as a recycled material for various applications including bitumen modification, concrete construction, and furniture production ( Abu-Saleem et al., 2021; Rafiq & Al-kamaki, 2022). Recycling PET waste as aggregates in concrete offers significant environmental benefits, primarily through substantial waste reduction by diverting large quantities of non-biodegradable PET from landfills and the natural environment, helping mitigate plastic pollution ( Sau et al., 2023; Tejaswini et al., 2022). Repurposing PET into construction material also reduces the demand for virgin aggregates, conserving natural resources ( Ponmalar, 2023). Additionally, energy savings are achieved by lowering the production of natural aggregates, which involves energy-intensive quarrying, crushing, and transportation powered mostly by fossil fuels that emit substantial CO _2_ and other greenhouse gases ( Vijerathne et al., 2024). This approach supports sustainability by addressing plastic waste, promoting sustainable construction, conserving energy, and reducing greenhouse gas emissions, thereby contributing to a circular economy.

Experimental studies have demonstrated that recycled PET fibres contribute to improved flexural response and crack control under bending loads, particularly when optimised in geometry and content ( Meza & Siddique, 2019). Mechanical optimisation research further shows that tailored combinations of fibre length and proportion can produce statistically significant enhancements in toughness, energy absorption, and serviceability performance ( Meza et al., 2021). Additionally, recycled PET fibres exhibit distinct anisotropic and bond characteristics that influence their reinforcement efficiency, enabling effective stress transfer and improved crack arresting mechanisms in concrete matrices ( Meza de Luna & Shaikh, 2020). Consequently, recycled PET fibre-reinforced concrete is being considered for structural and non-structural applications including slabs, pavements, precast elements, and repair works where enhanced ductility, crack resistance, and sustainability are desired.

PET plastic can be efficiently recycled by utilizing trusted technologies such as mechanical recycling, chemical hydrolysis, and melt processing, among others ( Grigore, 2017; Pavel & Awaja, 2005). Recycled PET is typically used in construction as aggregates for cement-concrete and as modifiers for asphalt concrete mixes ( Askar et al., 2023). PET waste is first sorted and separated from other types of plastic waste, and thoroughly washed to remove residual contaminants such as labels, adhesives, and organic matter ( Muringayil Joseph et al., 2024). Recycling waste PET for cement concrete applications involves grinding into small flakes or granules of uniform particle size, melting, and extruding into aggregates with consistent morphology or melting and crushing into aggregates with varied morphology ( C. K. A. Uche et al., 2024). For asphalt modification, PET flakes or granules are blended with hot asphalt binder in a mixer or extruder at high temperatures with mechanical agitation to ensure uniform dispersion of PET in the asphalt ( Agha et al., 2023; Fahmy et al., 2024). Compatibilisers or adhesion promoters may be added to improve the bonding between PET and asphalt ( Masri, T. D. K. A. et al., 2022). The mechanical properties of recycled plastics have led to their use in several applications. Their low density, ease of processing, moderate chemical resistance, particularly for thermal and electrical insulating materials, and cost-effectiveness relative to other recycled materials contribute to their versatility ( Sulyman et al., 2016). PET is easier to recycle than other thermoplastic polymers. Thus, replacing concrete aggregates with PET and using PET fibres as concrete reinforcement to increase their tensile strength can address the disposal problem while preserving the natural environment ( Foti, 2013; Qaidi & Al-kamaki, 2020).

Concrete is a material composed of aggregates (fine and coarse) held together by a bonding cement that hardens (cures) over time ( Gagg, 2014). Globally, concrete is the most utilized building material ( Amran et al., 2020). Concrete is inexpensive, durable, strong, easy to handle, and can be manufactured in any form or dimension. It is the most utilized building material, and only water is consumed more than concrete ( Basha et al., 2016; Faraj et al., 2020). Lightweight concrete (LWC) is a form of concrete composed of either a lightweight aggregate or an expanding agent ( Lotfy et al., 2016). Lightweight concrete for structural applications has a density in the range of 1400 to 2000 kg/m ^3^, while the density of normal weight is a minimum of 2000 kg/m ^3^ ( Thomas & Bremner, 2012). The aggregates used in structural lightweight concrete (S.L.W.C.) are at the other end of the scale and are typically composed of pumice, scoria, expanded shales, clays, slates, and slags. By definition, the minimum compressive strength is 17.0 MPa, the majority of structural lightweight aggregates may produce concrete with compressive values higher than 35.0 MPa ( American Concrete Institute, 2014). Lightweight concrete has similar strengths to regular concrete, although typically 25% to 35% lighter than regular concrete ( Cavalline et al., 2017). Given its ability to combine sufficient strength with low structural weight, structural lightweight aggregate concrete is a valuable and adaptable material in modern construction. Prestressed or precast elements of all kinds, bridges, offshore oil platforms, multistory building frames and floors, and many other diverse applications are among its numerous and varied uses ( American Concrete Institute, 2014; K. Thienel et al., 2020). Structural lightweight concrete had a global market worth USD 37.2 billion in 2018. Its growth projection was USD 56.7 billion by 2016. Thus, the rising global demand for structural lightweight aggregates has been demonstrated ( Reports and Data, 2020).

Lightweight concrete mixtures are designed by combining components in a technically and economically sound method; these components typically include water, aggregates, cement, and chemical or mineral admixtures. This allows the wet and dry phases of concrete to develop the required properties ( American Concrete Institute, 2014). Owing to the significant influence of the lightweight aggregates used, the proportioning of lightweight concrete differs fundamentally from that of regular-weight concrete. ( Tajra et al., 2019). Hardened cement paste typically has greater strength than lightweight aggregate filler material; thus, normal-weight concrete has a higher compressive strength than lightweight aggregate concrete with the same water/cement ratio ( Aşik, 2006). The maximum strength of the lightweight concrete is determined by the quality of the selected coarse lightweight aggregate. The strength limit is the point at which the lightweight aggregate becomes critical, and the strength of the matrix becomes subordinate to the potential strength of the lightweight concrete ( K.-C. Thienel & Sposito, 2017). The binder concentration and water-to-binder ratio in lightweight concrete are less important above this strength limit. Nonetheless, they must be carefully selected because they also affect the durability of lightweight concrete ( K. Thienel et al., 2020).

AC1 211-98 ( ACI Committee 211, 1998) recommends a mix design for structural lightweight concrete based on the volume or weight of the components. Several trial-and-error iterations are necessary to obtain these specific data. Moreover, tables and graphs that are currently available were created using restricted test parameters. EuroLightCon ( EuroLightCon, 2000) suggested a rational mix-design approach for lightweight aggregate concrete. A thorough analysis was conducted on the absorption properties of two commercially available aggregates: Lytag (sintered fly ash) and Liapor (expanded shale). Forty per cent of the aggregate volume was set aside for the sand volume. This process is only applicable to water–cement ratios from 0.25 to 0.35. Bogas and Gomes ( Bogas & Gomes, 2013a) presented a straightforward procedure based on a biphasic model for producing structural concrete from lightweight aggregates. The mortar component of the concrete and the characteristic strength of the lightweight aggregate were included as mix design factors. The other values were calculated by assuming the volumes of the paste and coarse aggregates. Yang et al. ( Yang et al., 2014) developed an initial mix-proportioning approach for Structural Lightweight Concrete (SLWAC) using regression analysis. A total of 347 data points were considered in the investigation, the majority of which were from clay lightweight aggregates or expanded fly ash. The dry density and absolute volume of concrete were considered as boundary conditions in this process. However, this approach lacks a clear definition of the absorption criterion. Nadesan and Dinakar ( Nadesan & Dinakar, 2017) presented a straightforward and trustworthy technique for designing lightweight concrete that uses round-shaped, sintered fly ash lightweight particles. The established link between the 28-day compressive strength of concrete and various mixture parameters was used to guide the proportioning process.

Lightweight concrete having compressive strengths up to 34.5 MPa has regularly been incorporated in commercial construction since the early 1930s. The strength of regular-weight concrete is determined by the strength of the mortar matrix. Thus, the concrete and matrix compressive strengths are uniformly correlated. The water/cement ratio and standard compressive strength of cement are factors that define the strength of a mortar ( K. Thienel et al., 2020). The load-bearing capability of the aggregate and the interface between the aggregate and cement paste become the limiting elements in strength growth when the design loads are closer to and above the strength limits of the cement paste matrix. Therefore, the strength of lightweight concrete construction depends entirely on lightweight aggregates. As a result, the compressive strength of the matrix may be greater than that of concrete ( Bogas & Gomes, 2013b). The point above which the lightweight aggregate strength capacity determines the strength of lightweight concrete is known as the strength limit ( Faust, 2003). Every aggregate has a strength ceiling, and for lightweight aggregates, the strength ceiling can usually be raised by lowering the maximum size of the coarse aggregate ( American Concrete Institute, 2014). Since compressive strength is considered a good indicator of other mechanical properties of lightweight concrete, such as tensile and flexural strength, it is important to examine the variables influencing compressive strength, particularly the type of lightweight concrete and lightweight aggregate utilized.

Natural resources used to make concrete have been overused and depleted as a result of increased urbanization and development over time ( Bhardwaj & Kumar, 2017). Studies have revealed that adding plastic particles to concrete results in strengths that are on par with those of ordinary concrete, making it appropriate for use in building applications ( Hussain, 2021; C. K. A. Uche et al., 2023). PET aggregates are typically produced by thermal extrusion, mechanical shredding/grinding, or a combination of both methods ( Schilive et al., 2021), ( C. K. A. Uche et al., 2023). Uche et. al. ( C. K. A. Uche et al., 2023) published a review of the production of lightweight concrete incorporating PET aggregates. Their results showed that PET aggregates have been widely used in structural lightweight concrete, with positive trends observed in compressive strength up to 20% and positive responses shown in tensile and flexural strength up to 10% when PET aggregate replacement was made. The introduction of PET aggregates led to an increase in water absorption, which is an indicator of concrete durability. Bamigboye et al. ( Bamigboye et al., 2022) conducted tests on concrete that partially or completely substituted natural coarse aggregates with recycled waste polyethylene terephthalate (PET). They noticed that the workability of concrete improved with an increase in the number of PET coarse aggregates. Regarding the compressive and tensile strengths, 20% PET substitution achieved the desired results for concrete grade 20. The thermal analysis results showed that the 100% PET sample experienced three transition phases. Research by ( Chong & Shi, 2023), reported that PET plastic works better in concrete when used as a fine aggregate substitute than when used as a coarse aggregate replacement. Further observation reveals that concrete with PET plastic added as a fine aggregate with up to 30% replacement can have a compressive strength that is adequate for structural applications.

Previous investigations into the incorporation of polyethylene terephthalate (PET) aggregates in concrete have primarily concentrated on their use as partial replacements for normal-weight aggregates, employing conventional mix design methodologies suited for normal-weight aggregates. Such approaches have yielded marginal structural performance and negligible waste valorisation potential ( Chong & Shi, 2023; C. K. A. Uche et al., 2023), leading to the conclusion that PET aggregates offer limited potential in structural concrete applications. However, this perspective overlooks critical material classification regarding the physical and mechanical characteristics of PET aggregates produced using the thermal/mechanical method, which align more closely with those of lightweight aggregates than with those of normal-weight aggregates ( C. K. A. Uche et al., 2024). Drawing from this alignment, a significant research gap exists in the application of PET aggregates within the conceptual and methodological frameworks of structural lightweight concrete. This study aims to design and evaluate the performance of mix designs for structural lightweight concrete with recycled (PET) lightweight aggregates as a complete replacement for conventional lightweight aggregates. The specific objectives are to: (i) develop experimental mix proportions using the factorial method; (ii) evaluate the performance with focus on fresh density, dry density, workability, compressive strength, splitting tensile strength, and water absorption. (iii) Demonstrate the suitability per standard performance criteria for structural lightweight concrete, and (iv) establish empirical relationships among the evaluated performance indices through statistical regression models. This approach not only aligns the material with its proper aggregate classification but also establishes a sustainable pathway for valorising PET waste in load-bearing applications.

## 2. Materials

### 2.1 Polyethylene terephthalate waste

Polyethylene terephthalate waste was collected from business and household waste bins around Ishaka Town, Western Uganda. These bottles were made of polyethylene terephthalate (PET), which is suitable for packaging food and drinks. The covers, labels and other non-PET plastic were removed and the bottles were shredded and thoroughly cleaned with water and mild detergent to remove impurities. The shredded PET were allowed to dry in open air without applying heat to remove moisture.

### 2.2 Calcium Hypochlorite (Ca (ClO) _2_)

Calcium hypochlorite (Ca (ClO) _2_) with the brand name ‘Cal-Hypo’, purchased from Hubei Xingfa Chemicals Group Co. Ltd. Hubei, China, was used in this study. It contains calcium ions (Ca ^2+^), hypochlorite ions (ClO ^-^), and water molecules. Calcium hypochlorite is widely used as a bleaching agent, disinfectant, and sanitizer.

### 2.3 Cement

The cement used in this study was 42.5N Portland Pozzolana Cement. It was obtained from the Tororo Cement Factory in Tororo, Uganda. This conforms to the US EAS 18-1:2017 ( Uganda National Bureau of Standards, 2017) and ASTM C150/C150M – 22 ( ASTM International, 2022). The physical and chemical properties of the cement are presented in Table 1.

**Table 1.** Physical and chemical properties of multipurpose 42.5N cement.

Properties	Standard requirement US EAS 18-1:2017
Physical properties	
2-day compressive strength	≥10MPa
28-day compressive strength	≥42.5MPa≤62.5MPa
Initial setting time	≥60mins
Soundness (expansion)	≤10mm
Chemical properties	
SO _3_	≤3.5%
Specific gravity	2.8 – 3.1
Boiling/melting point	>1000°C
Freezing point	None, solid
Viscosity	None, solid
pH	pH of wet cement, 12 – 14

### 2.4 Superplasticizer

A superplasticizer (SP) with the brand name “Sikament NNG KE, manufactured in Kenya, was used for this study. This conforms to ASTM C494 ( ASTM International, 2015) for types D and G. The physical and chemical properties of the superplasticizer are shown in Table 2.

**Table 2.** Physical and chemical properties of superplasticizer.

Properties	Description
Colour	Dark brown liquid
Composition	Based on naphthalene formaldehyde sulphonate
Density	1.21 ± 0.02 g/cm ^3^
pH	8.0 ± 1.0
Total chloride ion content	<0.1%

### 2.5 Water

The source of the clean water was the Materials Laboratory of the Department of Civil Engineering, Kampala International University, Western Campus, Ishaka, Uganda. This conforms to US EAS 12: 2014 ( Uganda National Bureau of Standards, 2014).

### 2.6 PET aggregates

**Figure 1.**
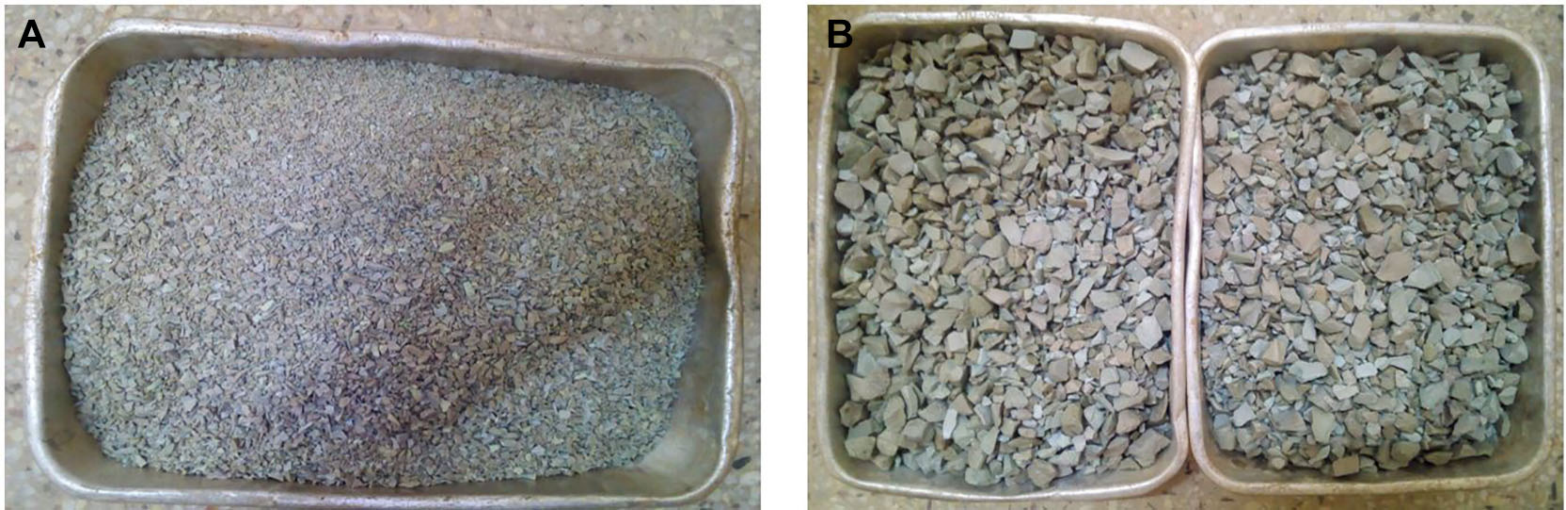
(a) Fine PET aggregates. (b) Coarse PET aggregates.

**Figure 2.**
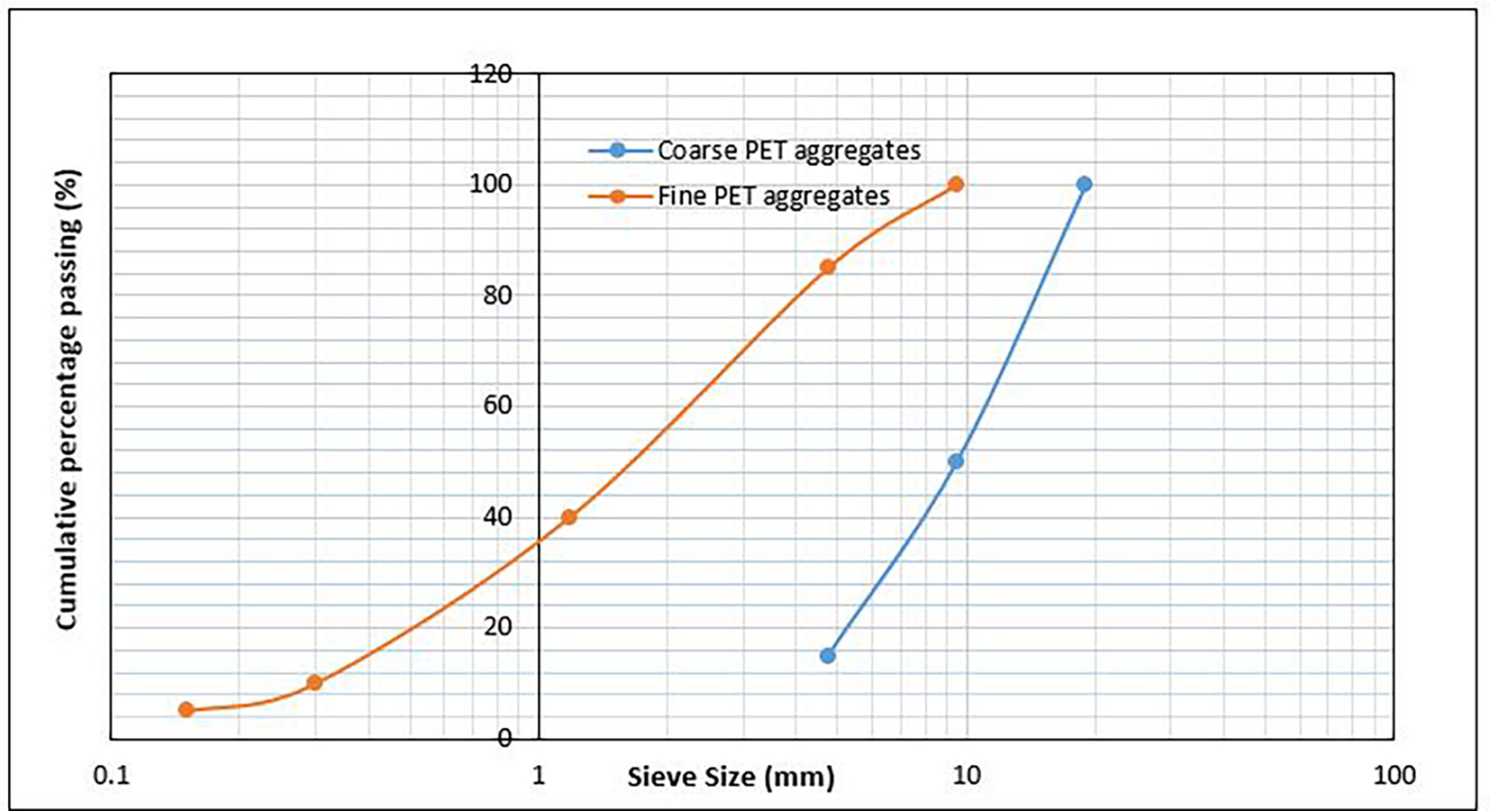
Particle size gradation of PET aggregates.

PET aggregates were produced following the thermal/mechanical procedure described by ( C. K. A. Uche et al., 2024). After mechanical, morphological and intrinsic characterisation, the authors in ( C. K. A. Uche et al., 2024) concluded that the aggregates are suitable for structural lightweight concrete. The PET aggregates were of heterogeneous sizes and shapes ( Figure 1a and 1b) and were graded according to ASTM C330 ( ASTM International, 2009) specifications for lightweight aggregates as shown in Table 3. The grading curves for the coarse and fine PET aggregates are shown in Figure 2, and the morphological, intrinsic, and mechanical properties of the PET aggregates are listed in Table 4.

**Table 3.** Particle size gradation of fine and coarse aggregates from PET.

PET Coarse aggregates		PET Fine aggregates (Fineness modulus = 2.6)	
Sieve size (mm)	Amount passing (%)	Sieve size (mm)	Amount passing (%)
19	100	9.5	100
9.5	50	4.75	85
4.75	15	1.18	40
-	-	0.3	10
-	-	0.15	5

**Table 4.** Mechanical, morphological and intrinsic properties of the PET aggregates ( C. K. A. Uche et al., 2024).

Mechanical properties	
Compressive strength	50 MPa
Aggregate crushing value	37%
Ten per cent fines value	71KN
Aggregate impact value	24%
Aggregate abrasion value	20%
Morphological Properties	
Flakiness index	26%
Elongation index	16%
Particle index	13
Intrinsic Properties	
Particle density	1330 kg/m ^3^
Bulk density	715 kg/m ^3^
Water absorption	0.445%

## 3. Experimental methods

The experiments were performed at the Materials laboratory of the Civil Engineering Department, Kampala International University, Western Campus, Ishaka, Uganda.

### 3.1 Chemical treatment of aggregates from PET

**Figure 3.**
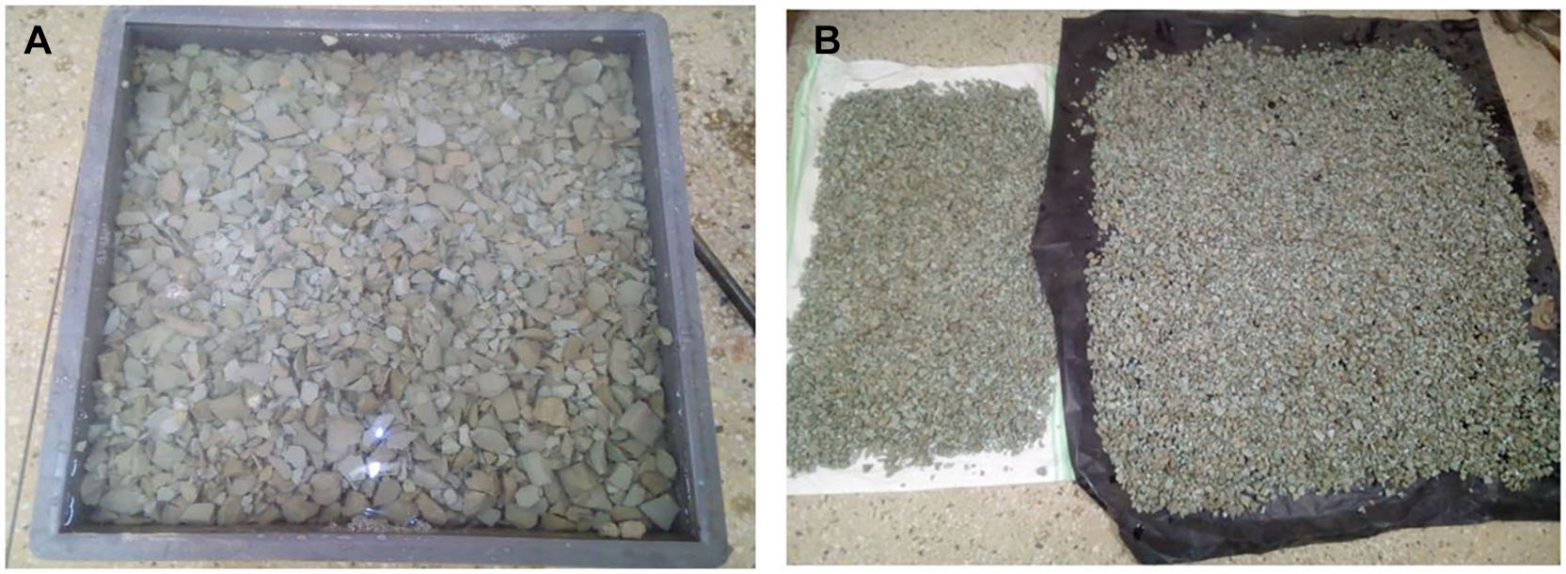
(a) PET coarse aggregates during chemical treatment. (b) Airdrying of chemically treated PET coarse aggregates.

The aggregates were subjected to chemical treatment using the procedure described previously ( Lee et al., 2019). This was performed to modify the surface of the aggregates to improve mechanical interlacing and chemical affinity for cement. The PET coarse aggregates were soaked in 5wt (Ca (ClO) _2_) for 24 h. The chemically treated PET aggregates were spread and air-dried to ensure that there was no residual solution on the surface. Figures 3a and 3b show the PET coarse aggregates formed during chemical treatment.

### 3.2 Specification of key design factors

The water/cement ratio for structural lightweight concrete is between 0.3 and 0.45 ( N. N. Hilal et al., 2021; Kim et al., 2020; Rodacka et al., 2023; Sahoo et al., 2022). In this study, the lower and upper limits of the water–cement (w/c) ratio were selected as 0.40 to 0.45 to balance strength, workability, and durability. This range was chosen based on its proven ability to meet the minimum compressive strength requirements for structural lightweight concrete (≥17 MPa), as recommended by ( Haque et al., 2004). Additionally, maintaining the w/c ratio within this range supports the development of a dense cement matrix, which improves the interfacial bond with PET aggregates and limits microcracking under load ( Bhise, 2022; Juki et al., 2013; Rahmani et al., 2013). The upper limit of 0.45 was adopted to ensure sufficient workability for effective mixing and compaction, while the lower limit of 0.40 was used to control capillary porosity, thereby enhancing resistance to permeability, shrinkage, and long-term durability concerns ( Badogiannis et al., 2021; Glas et al., 2015). This leads to the computation of other key design parameters from the mixture proportioning equations of ( Chandra & Berntsson, 2002).

**3.2.1 The volume of cement paste (v**_**p**_ **)**

The volume of the cement paste or binder used in the concrete was determined according to the principle of “Constant Paste Aggregate Volume”. A value of 30% that is 0.3 m ^3^ of cement paste per m ^3^ of concrete, was adopted for this study.

**3.2.2 Cement content**

The cement content is obtained using the Eqn (1):

$$C=1000*\frac{v_{p}}{0.31+\left(w/c\right)}$$ (1)

where C is the cement content (kg per m ^3^ of concrete), *v*_p_ is the volume of cement paste in the concrete, and w/c is the ratio of water content to cement content.

**3.2.3 The volume of aggregates**

The volume of lightweight aggregates was calculated from Eqn (2):

$$v_{\mathrm{agg}}=1-\left(v_{\mathrm{cem}}+v_{w}+v_{\mathrm{air}}\right)$$ (2)

where *v*_agg_ is the volume of the lightweight aggregates, *v*_cem_ is the volume of cement, and *v*_w_ is the volume of water.

The volume of the lightweight coarse aggregates was considered as 60% of the total volume of the lightweight aggregates. Thus, the volume of the coarse aggregates was obtained from Eq. (3)

$$v_{\text{cagg}}=0.6v_{\mathrm{agg}}=0.6\left(1-\left(v_{\mathrm{cem}}+v_{w}+v_{\mathrm{air}}\right)\right)$$ (3)

Thus volume of lightweight fine aggregates was obtained from Eqn. (4)

$$v_{\text{fagg}}=\left(1-0.6\right)v_{\mathrm{agg}}=0.4v_{\mathrm{agg}}=0.4\;\left(1-\left(v_{\mathrm{cem}}+v_{w}+v_{\mathrm{air}}\right)\right)$$ (4)

where $v_{\text{fagg}}$ is the volume of fine aggregates and $v_{\mathrm{agg}}$ is the total volume of aggregates.

The key design factors as computed are displayed in Table 5.

**Table 5.** Key design factors for proportioning structural lightweight concrete mixture.

		Mixture components (kg/m ^3^ of concrete)			
	W/C ratio	Cement	Water	Fine PET aggregates	Coarse PET aggregates
Lower Limit	0.4	395	158	346	519
Upper limit	0.45	423	190	372	545

### 3.3 Experimental design of concrete mixture proportions

The mathematically independent variable (factorial) method of experimental design, as described by ( Simon, 2003), was used to design the mixture proportions. It was applied to develop experimental concrete mixes with full replacement of natural aggregates using fine and coarse PET aggregates. The ratio of two components is used as an independent variable in the factorial approach to reduce the q components of a mixture to q-1 independent variables. For concrete, the water/cement ratio is ideal as an independent variable. The foundation of the experiment in the case of q-1 independent variables was a 2 ^q-1^ factorial design. Several factors (variables) were used in this design, and they were set at two separate levels. Water, cement, fine aggregates, and coarse aggregates constitute the majority of the concrete mixtures. This system can be described using three independent factors or variables: x _1_ = water/cement ratio, x _2_ = fine aggregate portion, and x _3_ = coarse aggregate portion. Any measurable fresh or hardened feature of concrete, as defined, can be one of the dependent variables y _i_, sometimes referred to as the responses or performance criteria. The Stat-Ease Design Expert software (version 11) was used for the experimental design. The experimental mix design for the lightweight structural concrete is presented in Table 6.

**Table 6.** Experimental mix designs for structural lightweight concrete.

		Mixture components (kg/m ^3^ of concrete)			
Run	Water/cement ratio	Cement	Fine PET aggregates	Coarse PET aggregates	Superplasticiser
1	0.4	395	346	519	3.95
2	0.45	395	372	519	3.95
3	0.45	395	346	545	3.95
4	0.4	423	346	545	4.23
5	0.45	423	346	519	4.23
6	0.4	423	372	519	4.23
7	0.4	395	372	545	3.95
8	0.45	423	372	545	4.23

### 3.4 Batching, mixing and curing of concrete specimens

**Figure 4.**
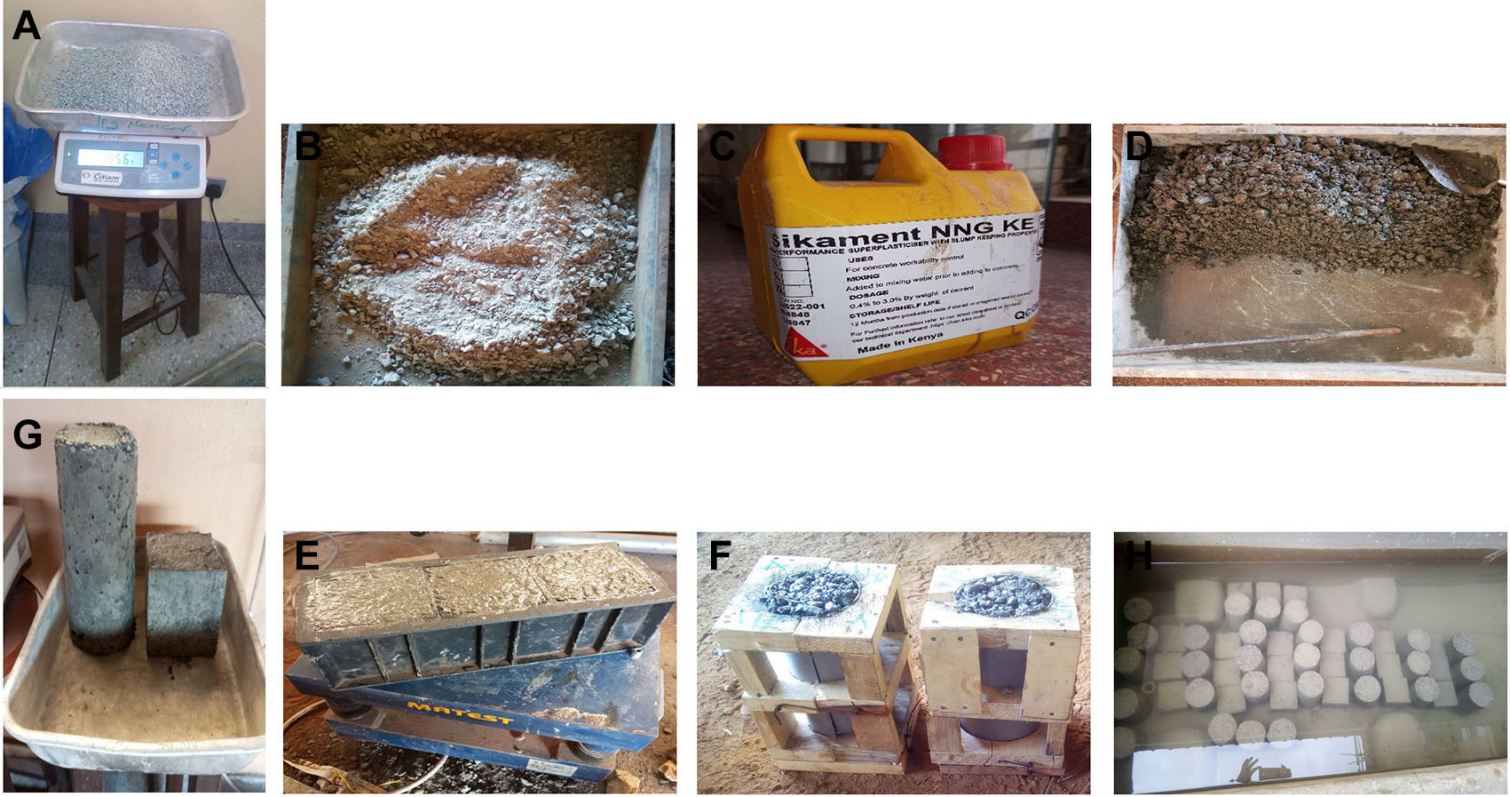
(a) Measuring of PET aggregates. (b) Mixing of fine and coarse PET aggregates with cement. (c) Superplasticizer. (d) Mixing of PET aggregates and cement with superplasticizer and water. (e) Compaction of fresh concrete using MATEST C183 vibrating table. (f) Cylindrical mould containing fresh concrete. (g) Hardened concrete specimens. (h) Concrete specimens in curing tank.

Batching and mixing of the designed concrete mixes were performed manually according to BS 1881 – 125:2013 ( British Standards Institution, 2013). The aggregates were weighed on a CITIZEN SSH 93 L weighing scale to an accuracy of ±1%, and the cement and water to an accuracy of ±0.5% for every batch of concrete. The superplasticizer was weighed at 1% of the cement content and added to the mixing water. 48 cubes (100 mm × 100 mm × 100 mm) and 24 cylinders (100 mm diameter × 200 mm length) were used in this study. The mould was filled with concrete and placed on a MATEST C183 vibrating table until the surface of the concrete was level with a smooth glossy finish. A jute bag was placed over the moulds containing concrete for 24 h. Subsequently, the hardened concrete specimens were removed and placed in a curing tank filled with water for 28 d. The mean temperature and humidity of the curing location is 20°C and 83% respectively. The batching, mixing, and curing processes are shown in Figures 4a– 4h.

### 3.5 Performance evaluation of concrete

**3.5.1 Workability**

**Figure 5.**
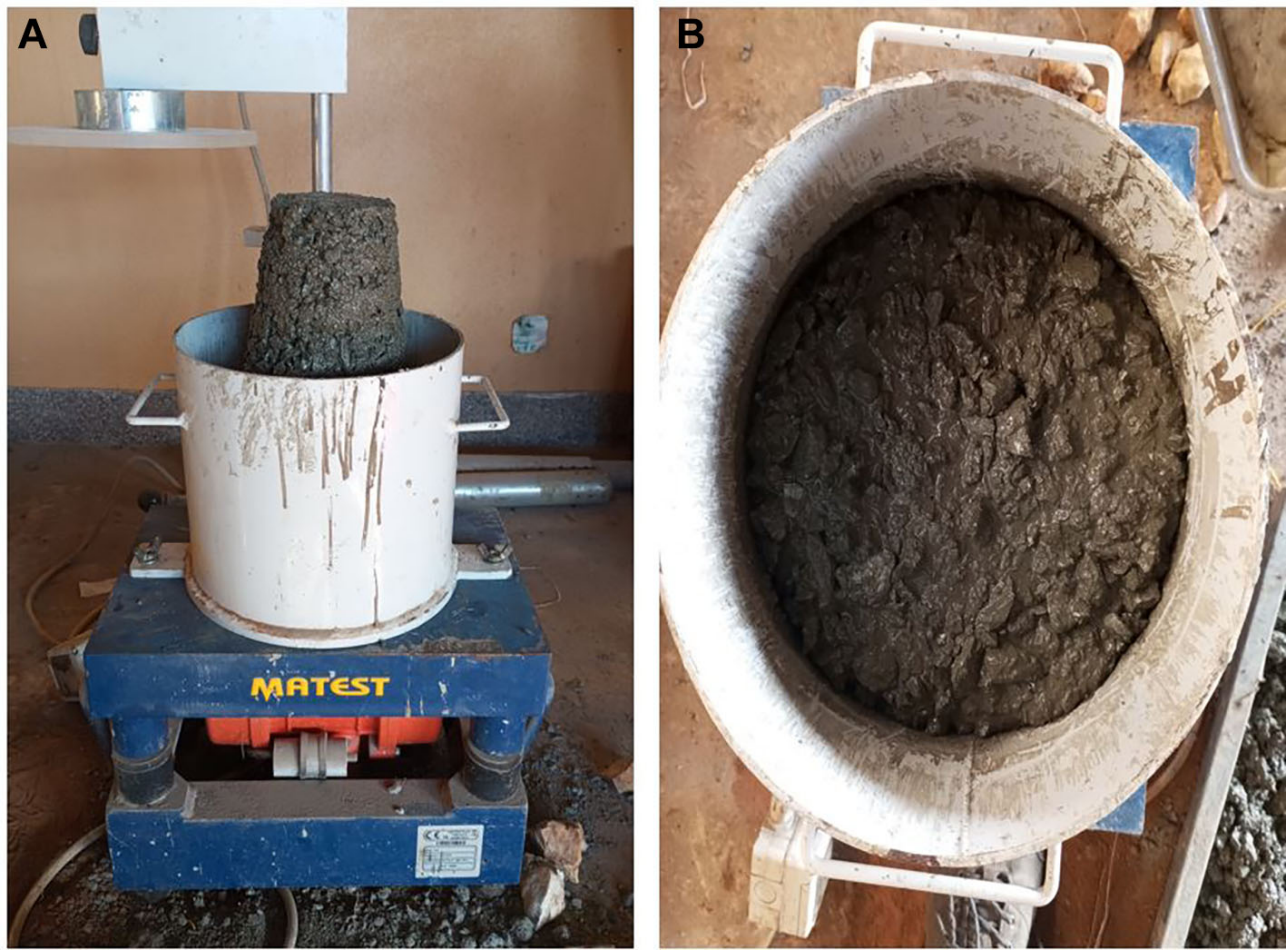
(a) Concrete slump cone in vebe test machine. (b) Surface of concrete after recording the vebe time.

The workability of the concrete was determined by the Vebe test using a MATEST C183 Vebe meter. This test was performed on fresh concrete following BS EN 12350 – 3:2019 ( British Standards Institution, 2019b). The compaction of the newly mixed concrete formed a slump cone. After lifting the cone away from the concrete, a clear disc was swung over its top and gradually lowered such that it touched the concrete. The vibrating table was turned on, and the Vebe time (s), which is the amount of time it takes for the lower surface of the transparent disc to fully contact the grout, was recorded. The procedure is shown in Figure 5a– 5b.

**3.5.2 Determination of fresh density of concrete**

The procedure for determining the fresh density of concrete is given in IS 1199 (Part-3) – 2018 ( Bureau of Indian Standards, 2018b). The fresh concrete was compacted using a vibration table into a calibrated, rigid, and watertight container, and then weighed. The volume (V) of the container was obtained through calibration. The empty container was weighed, and the mass was recorded as (m _1_) and the mass of the compacted concrete plus the container (m _2_) was also recorded. The density of the concrete was calculated using Eq. (5):

The density was calculated using Eq (5):

$$\rho_{f}=\frac{m_{2}-m_{1}}{V}$$ (5)

where D is the fresh density of the concrete (kg/m ^3^), m _2_ is the mass of the compacted concrete plus the container (kg), m _1_ is the mass of the empty container (kg), and V is the volume of the container (m ^3^).

The result of the density determination was expressed to the nearest 10 kg/m ^3^.

**3.5.3 Determination of hardened concrete density**

**Figure 6.**
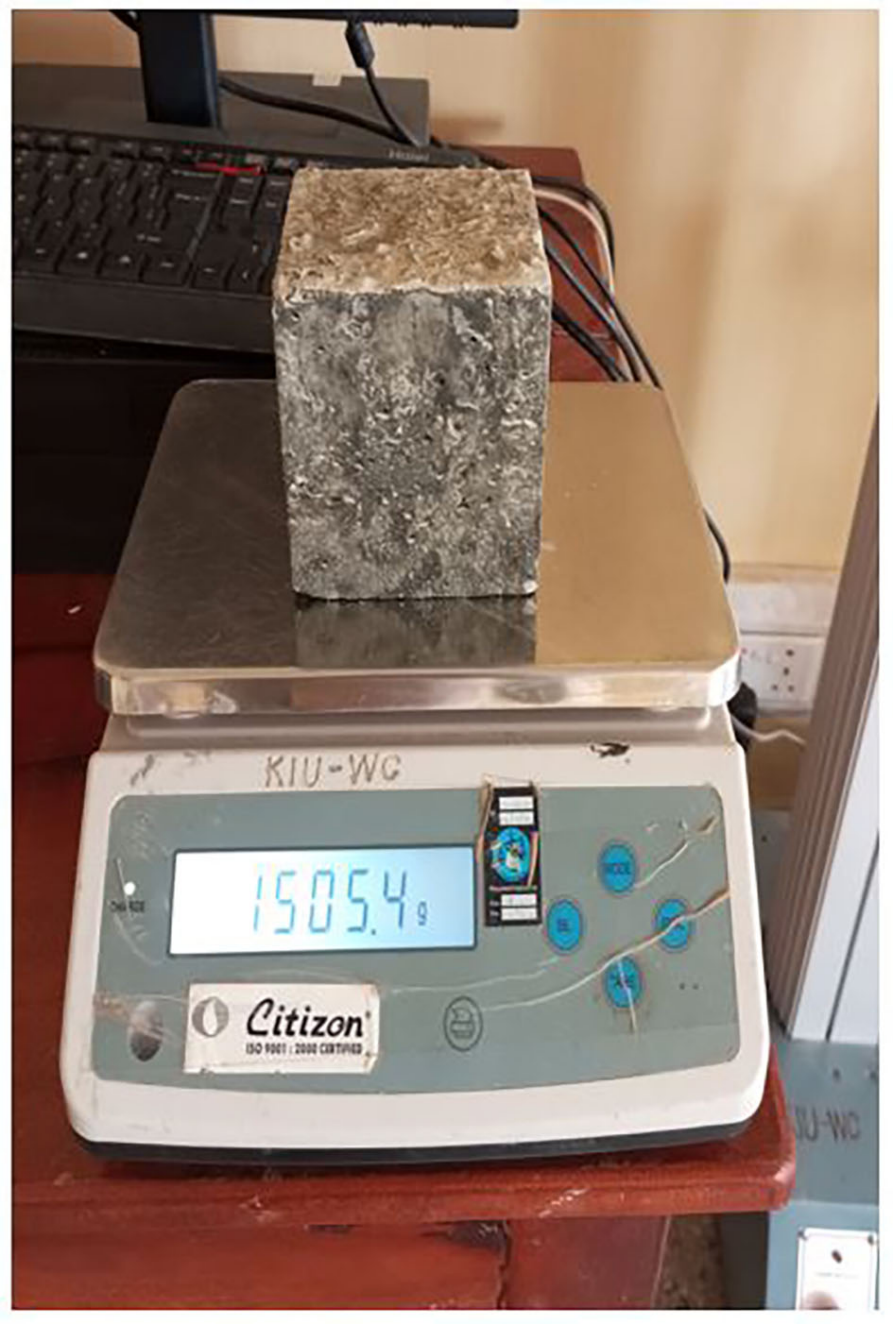
Hardened cubic concrete specimen.

The hardened density of the concrete ( Figure 6) was determined after 28 d of curing following BS EN 12390-7:2019 ( British Standards Institution, 2019c). The volume was calculated from actual measurements made on the specimen in m ^3^, rounded to four significant Figures, or by using designated dimensions (cubes only), where the volume of the cube was calculated in m ^3^, expressed as three significant figures.

The density was calculated using Eqn. 6

$$\rho_{d}=\frac{m_{d}}{V}$$ (6)

where $\rho_{d}\;$ is the density (kg/m ^3^), m _d_ denotes the mass of the dry specimen (kg), and V denotes the volume (m ^3^).

The value of density determined was rounded up to the closest 10 kg/m ^3^.

**3.5.4 The compressive strength of concrete**

**Figure 7.**
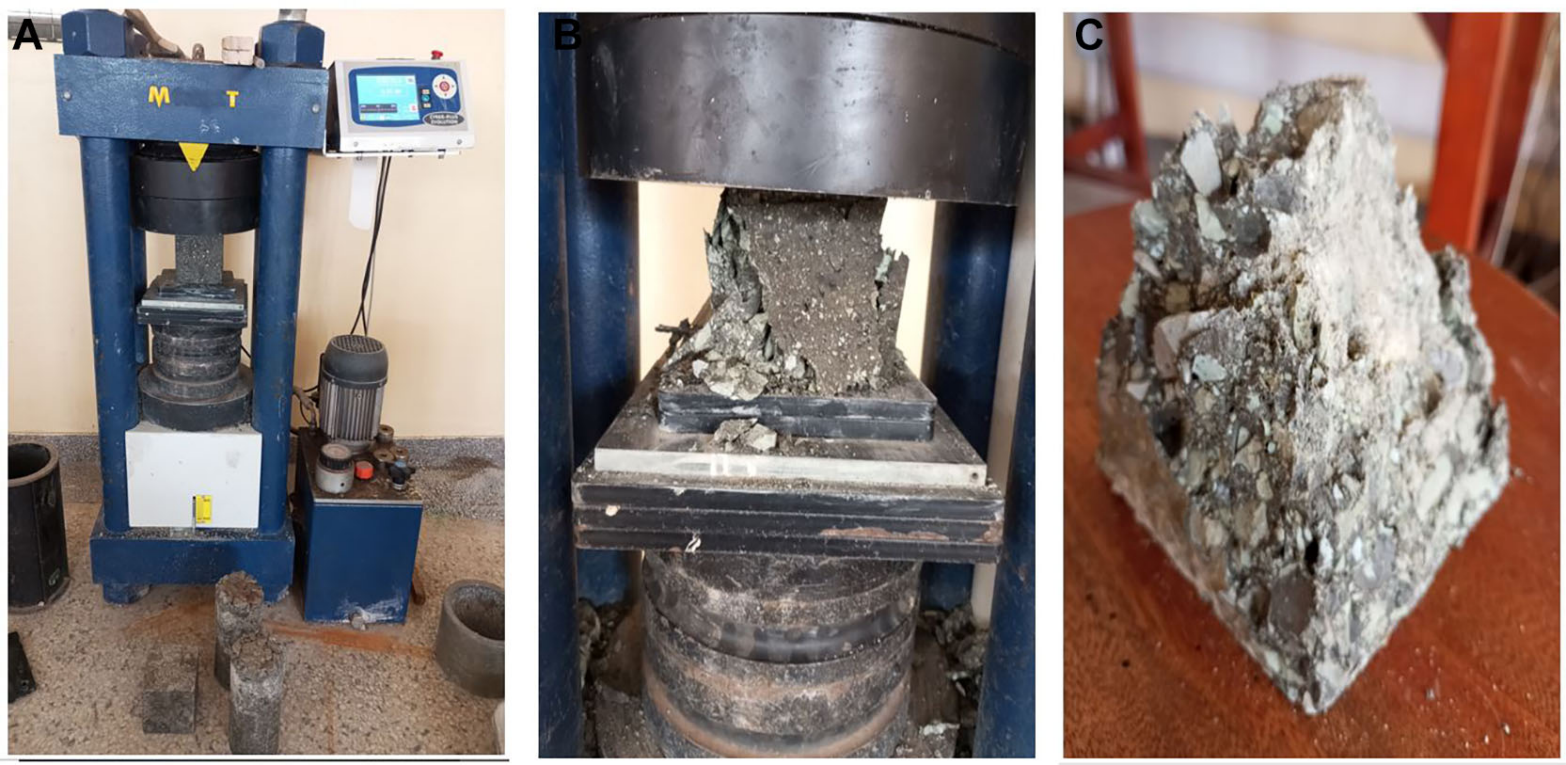
(a) Compressive strength testing. (b) Concrete specimen after failure. (c) Failed cubic specimen.

This compressive strength test was performed following BS EN 12390-3:2019 ( British Standards Institute, 2019). A MATEST CO89PN140 compressive-strength testing machine was used to apply a steady direct load at a rate of 0.25 *Nmm* ^−2^ *s* ^−1^ to the concrete specimen until it reached the failure point. The maximum supported load was used to compute the compressive strength of the concrete specimens. The test procedure is illustrated in Figures 7a– 7c.

The compressive strength was calculated using Eqn (7):

$$f_{c}=\frac{F}{A_{c}}$$ (7)

Where, F is the maximum load at failure (N), A _c_ is the cross-sectional area of the specimen (mm ^2^) that receives the compressive force, and f _c_ is the compressive strength (MPa) or (N/mm ^2^). It is necessary to express the compressive strength to the closest 0.1 MPa (N/mm ^2^).

**3.5.5 The splitting tensile strength of concrete**

**Figure 8.**
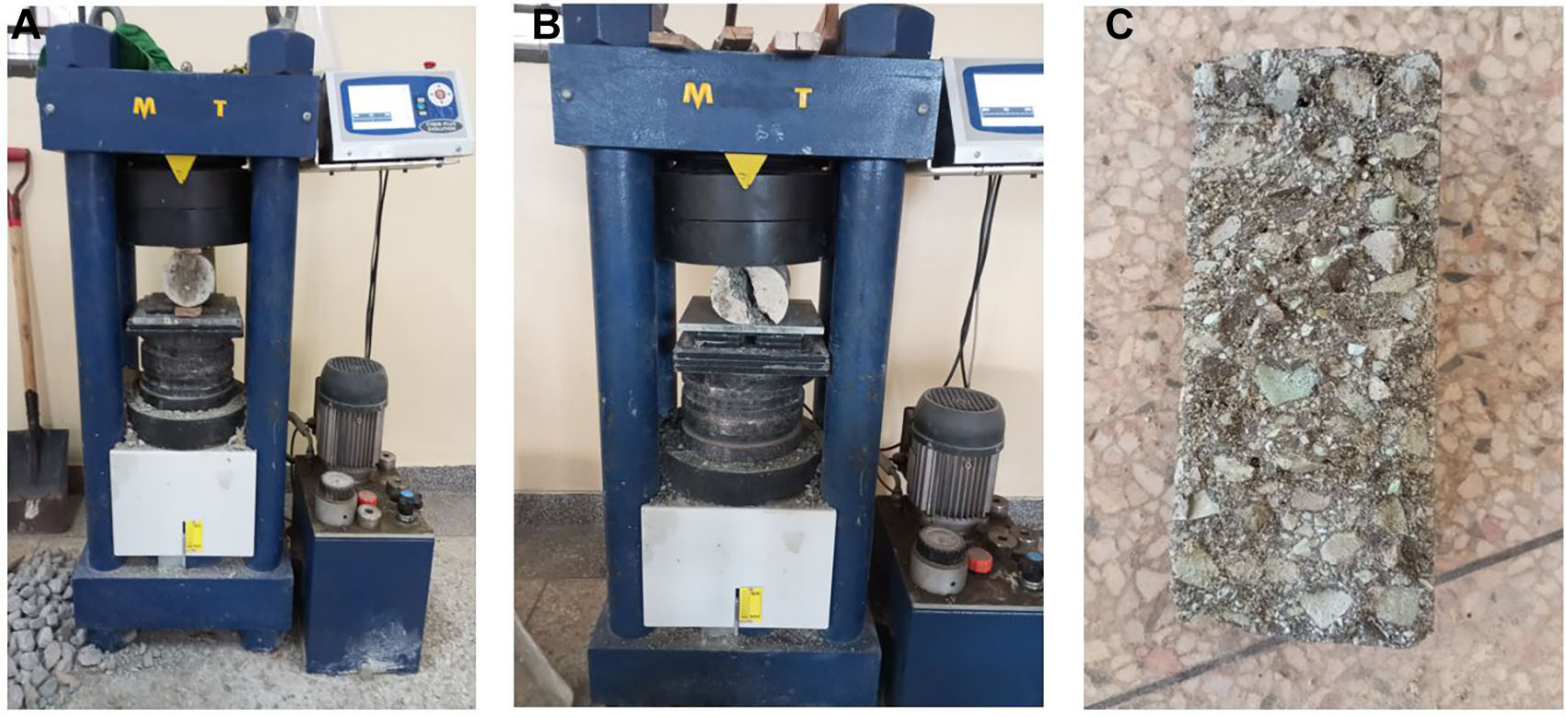
(a) Splitting tensile strength testing. (b) Concrete specimen after failure. (c) Failed cylindrical specimen.

This test was performed following BS EN 12390 – 6:2000 ( British Standards Institution, 2019a). A compressive force was applied lengthwise to a thin section of the cylindrical concrete specimen. The specimen failed under tension because of the orthogonal tensile force. The test procedure is illustrated in Figures 8a– 8c.

The splitting tensile strength was calculated using Eqn (8):

$$f_{\mathrm{ct}}=\frac{2\;x\;F}{\pi \;x\;L\;x\;d}$$ (8)

where *f*_ct_ is the tensile splitting strength (MPa) or (N/mm ^2^), *F* is the maximum load (N), *L* is the specimen’s line of contact length (mm), and *d* is the specified cross-sectional diameter (mm).

**3.5.6 Water absorption of hardened concrete**

**Figure 9.**
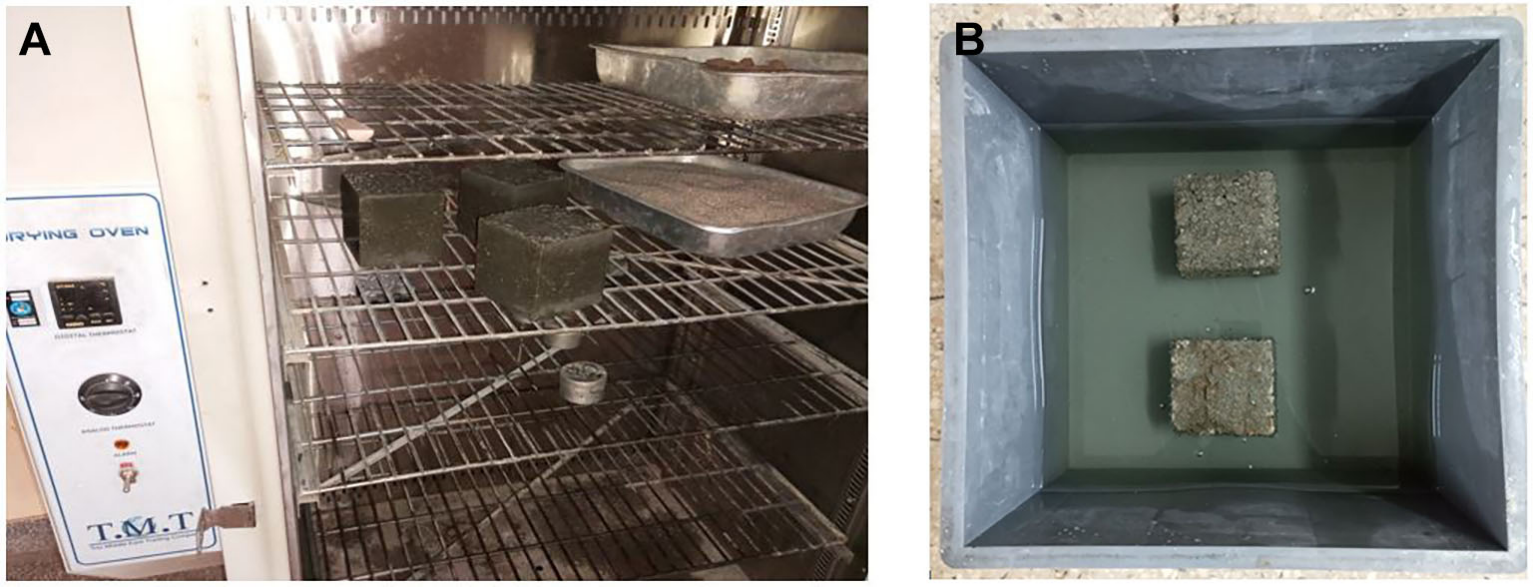
(a) Concrete specimens placed in the oven. (b) Concrete specimens immersed in water.

This test was performed following BS 1881-122:2011 ( British Standards Institution, 2011). After curing for 28 days, the concrete specimens were subjected to a water absorption test ( Figures 9a– 9b). The concrete specimen was placed in an oven, dried at a temperature of 105°C for 72 ± 2 h, and allowed to cool for 24 ± 2 h in an airtight vessel. The mass of each specimen was obtained and recorded (m _1_) immediately after cooling before it was immersed in a water tank with a water depth of 25 ±5 mm atop the specimen. The specimens were immersed in water for 30 ± 0.5 mins after which they were removed, and all excess water was removed from its surface using a dry, soft, and absorbent cloth. The mass of each specimen (m _2_) was recorded.

The water absorption was calculated using Eqn. (9):

$$\text{Water absorption}\;\left(\%\right)=\frac{m_{2}-m_{1}}{m_{1}}\;x\;100$$ (9)

where m _1_ is the mass of the dry concrete specimen (kg) and m _2_ is the mass of the soaked concrete specimen (kg).

## 4. Results and discussion

The results of standard tests on the experimental mix designs are displayed in Table 7.

**Table 7.** Results of standard tests on the experimental mix designs.

Mix	Fresh density (kg/m ^3^)	Vebe time (s)	Dry density (kg/m ^3^)	Std. dev.	Compressive Strength (MPa)	Std. dev.	Splitting Tensile Strength (MPa)	Std. dev.	Water Absorption (%)	Std. dev
1	1491	32	1492	6.7	16.3	0.15	1.4	0.04	7.4	0.2
2	1512	21	1537	5.4	16.5	0.06	1.2	0.12	9.8	0.55
3	1477	15	1500	6.4	15.2	0.36	0.99	0.02	4.66	0.29
4	1515	40	1536	2.3	16.1	0.09	0.84	0.06	9.49	0.4
5	1504	16	1523	5.5	14.8	0.08	0.92	0.05	10.16	0.02
6	1486	30	1509	3.0	15.6	0.0	0.86	0.07	4.74	0.2
7	1455	22	1490	7.9	14.1	0.02	1.1	0.11	6.4	0.09
8	1462	13	1495	9.3	15.4	0.1	1	0.13	6.5	0.52
Average	1488	24	1510		15.5		1.04		7.4	
Standard Deviation	22.2	9.5	19.4		0.81		0.19		2.21	
Coefficient of variation (%)	1.5	40	1.3		5		18		2	

The standard deviation values across the rows in Table 7, offer important insights into the consistency of each concrete mix regarding each measured property. The compressive strength results showed minimal variability (≤0.36 MPa); dry density values remained stable, with Mix 8 exhibiting the highest deviation (9.3 kg/m ^3^). Splitting tensile strength values were also consistent, apart from a slight increase in Mix 8 (0.13 MPa). Water absorption standard deviations varied more, ranging from 0.02% to 0.55%, with Mix 2 showing the highest inconsistency. Mix 5 demonstrated strong internal consistency, especially in water absorption (0.02%). Properties and mixes with low standard deviation indicate reliable and consistent mix preparation and testing, reflecting a well-compacted and uniform microstructure. Conversely, higher standard deviation values suggest potential issues with compaction or segregation, implying inconsistencies in interfacial bonding or pore structure. Overall, the standard deviation data supports the repeatability of results.

The values of the standard deviation for each test indicated that the experimental results were consistent and did not deviate considerably from the average. The coefficient of variation was obtained by dividing the standard deviation by the average strength value for each test. The coefficient of variation for the standard tests indicates that the results cluster closely around the mean and fall into the low category (0 – 20%), which implies consistency and stability. A notable exception is workability (vebe time), which exhibits a moderate spread around the mean, indicating a moderate level of variation.

The detailed table of concrete test results with standard deviation values is provided as a supplementary file.

### 4.1 The density of concrete

**Figure 10.**
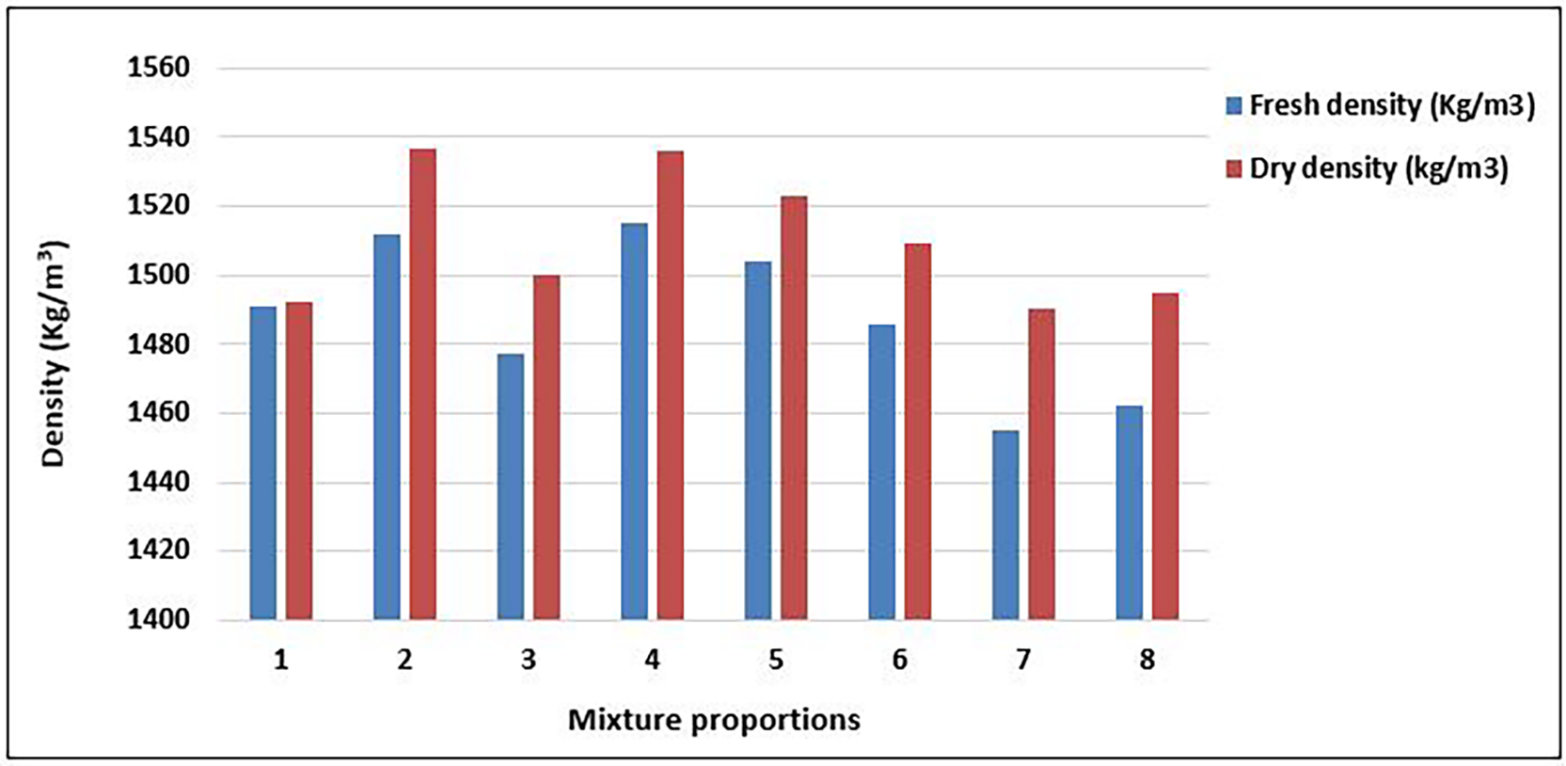
Values of density for concrete mixture proportions.

The density of concrete is a crucial factor influencing its mechanical properties, durability, and structural efficiency. The major determinant of lightweight concrete is its density. Concrete with oven-dry density in the range of 800 kg/m ^3^ and 2000kg/m ^3^ is regarded as lightweight concrete following BS EN 206:2013+A2:2021 ( British Standards Institution, 2021). The fresh density of concrete depends on the aggregate relative density, water demand, absorbed moisture content of lightweight aggregates, mixture proportions, and air content ( American Concrete Institute, 2014). Figure 10 displays the values of the fresh and dry densities of the concrete mix proportions after 28 days of curing.

As shown in Figure 10, the concrete produced from all mixture proportions satisfies the criteria for structural lightweight concrete ( American Concrete Institute, 2014; British Standards Institution, 2021). The range of the fresh concrete density was 1455 – 1515 kg/m ^3^ with an average value of 1488kg/m ^3^. Hardened concrete density was recorded between 1490 and 1537kg/m ^3^ with an average of 1510kg/m ^3^. These values fall under category D 1.6 (1400 kg/m ^3^–1600 kg/m ^3^) ( British Standards Institution, 2021). The concrete from mixes 2 and 4 had the highest values of both fresh (1512kg/m ^3^; 1515kg/m ^3^) and dry density (1536kg/m ^3^; 1537kg/m ^3^), whereas mix 7 had the lowest values in both fresh (1455kg/m ^3^) and dry density (1490kg/m ^3^). Mixtures 7 and 8, which had the highest quantity of aggregates, had the lowest fresh and dry densities, respectively.

In most of the mixture proportions, there was a significant increase between fresh and dry densities, with mix 1 having the lowest increase of 0.067% and mix 7 having the greatest increase of 2.41%. This is because the voids in the concrete matrix were filled with the products of the cement hydration reactions. The density is a factor that influences the mechanical properties of lightweight structural concrete. Structural lightweight concrete with a greater density tends to have fewer voids, less porosity, and higher strength, thus exhibiting greater durability ( Iffat, 2015). The density range here is relatively high for plastic-based concrete, indicating good compaction and effective matrix-aggregate bonding in certain mixes (e.g., Mixes 2, 4, 5). The concrete shows potential to be densified through proper proportioning of PET particle sizes and binder content, reducing common issues in PET concrete such as poor bonding and high porosity. Higher-density concrete generally has lower porosity, leading to improved durability (less permeability, shrinkage, and chemical ingress) ( Sánchez-Mendieta et al., 2024). Mixes with densities above 1520 kg/m ^3^ (2, 4, and 5) are likely to perform better in aggressive environments than those below 1480 kg/m ^3^ (Mix 3, 7). Optimizing material proportions to increase dry density enhances durability and prolongs the service life of PET-based SLWC.

Lightweight concrete has been produced using PET aggregates as a partial replacement for fine and coarse aggregates in various sizes and percentages. Studies with the replacement of fine aggregates with PET aggregates up to 50% recorded dry density in the range of 1771 – 1993 kg/m ^3^ ( Almeshal, I. et al., 2020; El-Nadoury, 2022; Kangavar et al., 2022; Qaidi et al., 2023). A replacement of fine aggregates with 70% and 90% PET aggregates resulted in a dry density of 1998 kg/m ^3^ and 1898 kg/m ^3,^ respectively, as observed by ( Hanuseac et al., 2020). When 50% of coarse aggregates were replaced by PET aggregates, by ( Islam et al., 2016) and ( Farah et al., 2024) the dry density was measured as 1980 kg/m ^3^ and 1770 kg/m ^3^ respectively. Nursyami & Zebua ( Nursyamsi & Zebua, 2017) experimented with 100% replacement of coarse aggregates and obtained a dry density of 1800 kg/m ^3^. Total replacement of natural aggregates with PET aggregates as in this study produces mid – range density class of lightweight concrete, unlike the high range densities of the reported studies. This result is similar to ( C. Uche et al., 2022), howbeit with a different mix proportion and aggregate size gradation.

Other researchers have produced lightweight concrete using entirely lightweight aggregates, but with different mixture proportions and varying outcomes. Palacios et al. ( Palacios et al., 2020) produced all-lightweight concrete with equilibrium density ranges of 1672kg/m ^3^ and 1692kg/m ^3^ using thermally expanded clay and calcined clay as coarse and fine aggregates, respectively. Pumice, expanded shale, and expanded clay aggregates have yielded all-lightweight aggregate concrete with lower dry density compared to PET aggregates, as contained in this study ( Nguyen et al., 2014). Majhi et al. ( Majhi et al., 2021) used sintered fly ash and fly ash cenosphere for all-lightweight concrete with a fresh density of 1589kg/m ^3^ and hardened density of 1203kg/m ^3^. The result of this study shows similarity to the cited works, showcasing the suitability of PET aggregates for structural lightweight concrete.

### 4.2 Workability of concrete

**Figure 11.**
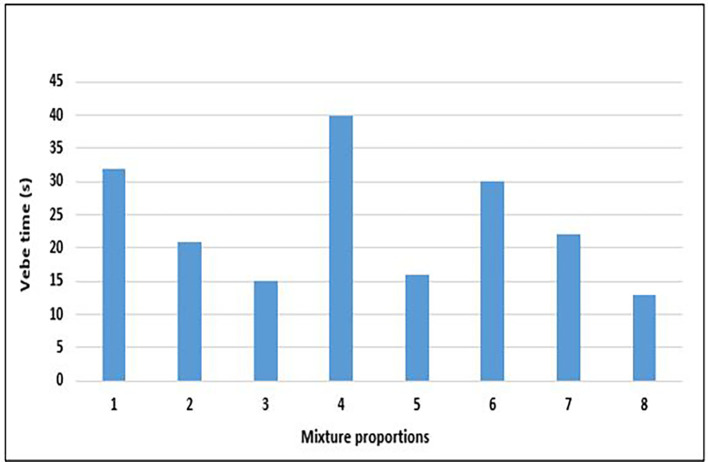
Results of vebe test for concrete mixture proportions.

The workability of fresh concrete refers to its fluidity and ability to be manipulated to fill the space within a receptacle prepared for it and to receive an adequate surface finish. The Vebe method is the most reliable test for lightweight aggregate concrete ( Rodacka et al., 2023). Structural lightweight concrete and normal-weight concrete exhibit markedly different workabilities. The results of the Vebe test on fresh concrete with different mix designs are shown in Figure 11.

**Figure 12.**
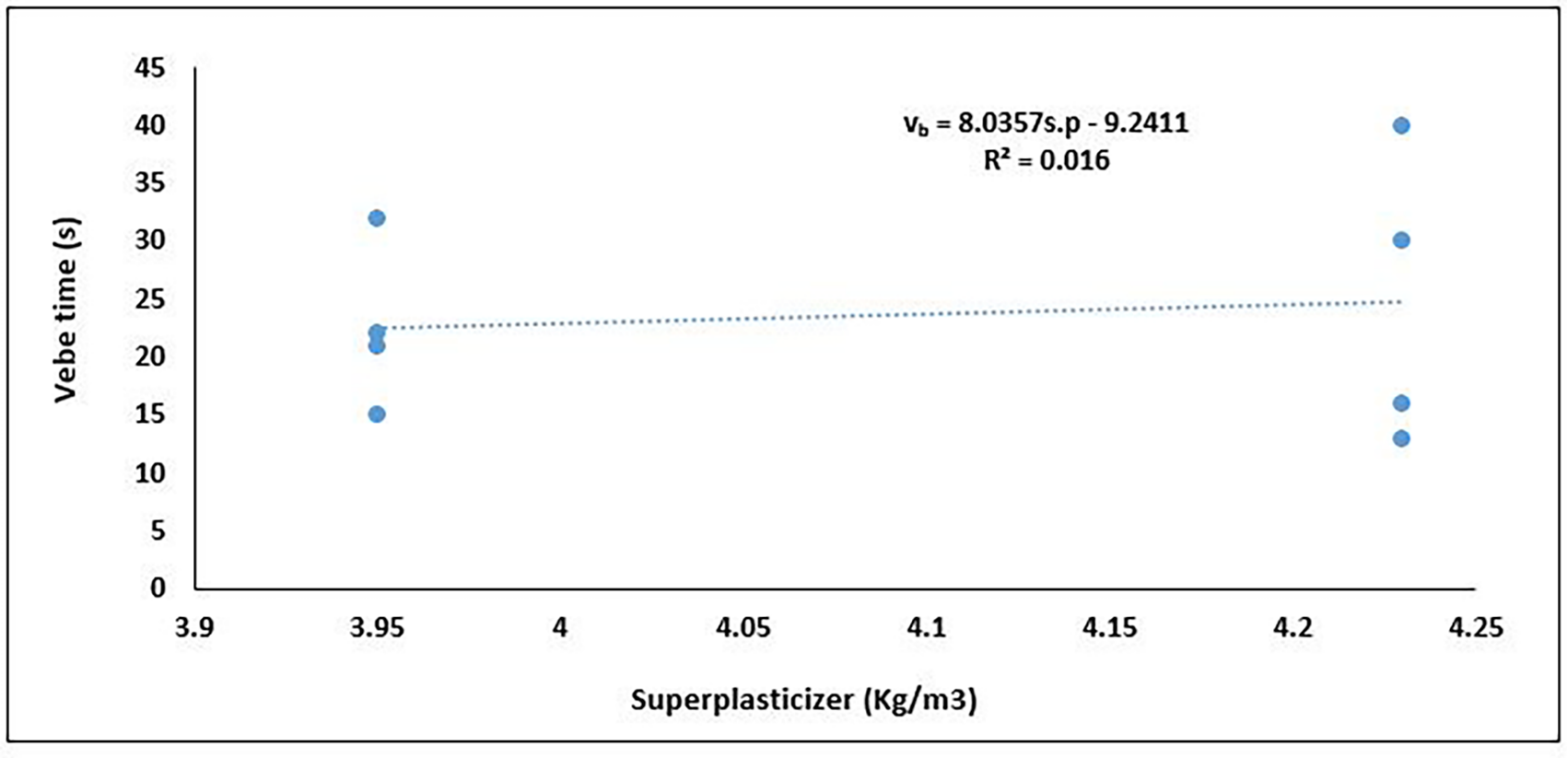
Relationship between vebe time and superplasticizer content.

All the mixture proportions had zero slump, and the range of vebe times for the concrete mixtures ranged from 13 to 40 s with an average value of 24 s ( Figure 11). According to I. S 1199 Part 2:2018 ( Bureau of Indian Standards, 2018a), this is concrete with a very dry consistency. This outcome was obtained despite the addition of a superplasticizer, which was expected to improve the workability of the concrete mixture. The highest and lowest workabilities were obtained for mixes 8 and 3, respectively. It was observed that mixes 3, 5, and 8, with a greater w/c ratio and greater total mass of aggregates, had higher workability values. This observation aligns with ( Islam et al., 2016), and is due to the hydrophobicity of PET lightweight aggregates compared to other lightweight aggregates, leading to greater fluidity in the fresh concrete matrix. In addition, workability can be regarded as dependent on the mass of fresh concrete, as it is measured by the fastest rate of ‘fall’ or ‘collapse’ of the concrete cone. From the results in Figure 11, the common denominator for mixes 4 and 6 with reduced workability is a low w/c ratio that translates into a higher cement and superplasticizer content. This observation is further highlighted in Figure 12, which shows the relationship between superplasticiser dosage and workability.

The incorporation of superplasticisers (as a percentage of cement content) in concrete mixtures is intended to enhance flowability and reduce internal friction, which should manifest as lower Vebe times—a direct indicator of improved workability ( Alsadey & Omran, 2022; Stenechkina, 2023). In the graph ( Figure 12), an unexpected positive trend between superplasticiser content and Vebe time for PET-based concrete is observed. Although the regression line suggests a slight upward slope, the very low coefficient of determination (R ^2^ = 0.016) confirms that superplasticiser content had a minimal response effect on the observed variations in Vebe time. This deviation from the expected trend may be attributed to interactions with PET aggregates, which are hydrophobic and lightweight, potentially disrupting the dispersion effect of the superplasticiser. The data indicate that in PET-lightweight concrete, factors such as PET aggregate content, packing density, or water-cement ratio may be influencing the rheological response more significantly, thereby diminishing the expected effect of superplasticizer dosage. The low R ^2^ value implies that an additive dosage of superplasticizer will have a marginal effect on the workability of PET-based lightweight concrete. Thus, superplasticizers should be used in synergy with viscosity-modifying agents (VMAs) or air-entraining agents—especially in mixes with hydrophobic aggregates like PET, which resist wetting and disrupt paste continuity. This shows the difference in behaviour between fresh, all-lightweight PET concrete and concrete made from other lightweight aggregates. An increase in cement (and by extension, superplasticiser) content generally improves the workability of lightweight aggregate concrete by enhancing paste volume and cohesion, thereby countering the high absorption and low density of lightweight aggregates ( Aboul-Nour & Zaghlal, 2020; Solak et al., 2018).

Lightweight aggregate concrete with varying extents of PET aggregates incorporation has been tested for their workability. These results are largely documented in ( C. K. A. Uche et al., 2023). The authors observed that the addition of PET aggregates had a significant negative impact on the slump of freshly mixed concrete. This observation was corroborated by previous studies ( Daisy Angel Priya et al., 2023; Qaidi et al., 2023). This is related to the lower fluidity caused by irregular PET aggregate morphologies. Other studies have presented results opposite to those mentioned above. The studies in ( Bachtiar et al., 2020) and ( Kayentao et al., 2023) observed a higher slump with an increase in PET aggregates across all concrete test specimens. Bamigboye et.al. and Ramakrishnan & Jegan, ( Bamigboye et al., 2021; Ramakrishnan & Jegan, 2023) observed an increase in the slump with PET replacement of up to 40% and 20%, respectively. Other experiments were performed by ( Lee et al., 2019) and ( El-Nadoury, 2022) to chemically modify the surface conditions of PET aggregates for concrete mixtures. These modifications reduced the slump value of concrete with such aggregates as the chemical modification caused the PET aggregates to have a rougher surface, thereby causing more friction at the interface of the aggregate particles and cement paste. The results of this study do not differ significantly from the available literature. A slump of 10 mm was recorded by ( C. Uche et al., 2022), with a water/cement ratio of 0.5, and the total substitution of normal-weight aggregates with heat-processed PET aggregates.

Crushed lightweight aggregates have a higher water absorption, which has a negative effect on workability ( K. Thienel et al., 2020). The workability of lightweight aggregate concrete was observed for different lightweight aggregates and mix proportions. Hilal et. al. ( N. N. Hilal et al., 2021) produced high-workability concrete with a 36% reduction in slump flow diameter compared with the control mix after a 50% substitution of coarse aggregates with walnut shells. The workability of the mixes with expanded shale and clay was 49% better than that of normal-weight aggregates. This was attributed to the lower angularity of these aggregates, as well as their lower specific gravity ( Mohammed et al., 2023). Slump values of 45 and 35 mm were obtained by ( Przychodzien & Katzer, 2021) for all-lightweight concrete mixtures comprising sintered fly ash and sintered fly ash combined with exfoliated vermiculite. All-lightweight concrete produced by ( Kim et al., 2020) using bottom ash aggregates reduced the workability by 23% compared to the maximum value obtained from a mixture with natural normal-weight fine and coarse aggregates. The workability of all-lightweight concrete made up of sintered fly ash as fine aggregates and fly ash cenosphere as coarse aggregates was recorded in the range of 90–95 mm ( Majhi et al., 2021). It was observed from the above-mentioned studies that lightweight aggregates with a largely spherical morphology had better workability than those with high angularity such as the PET aggregates in this study.

### 4.3 The compressive strength of concrete

**Figure 13.**
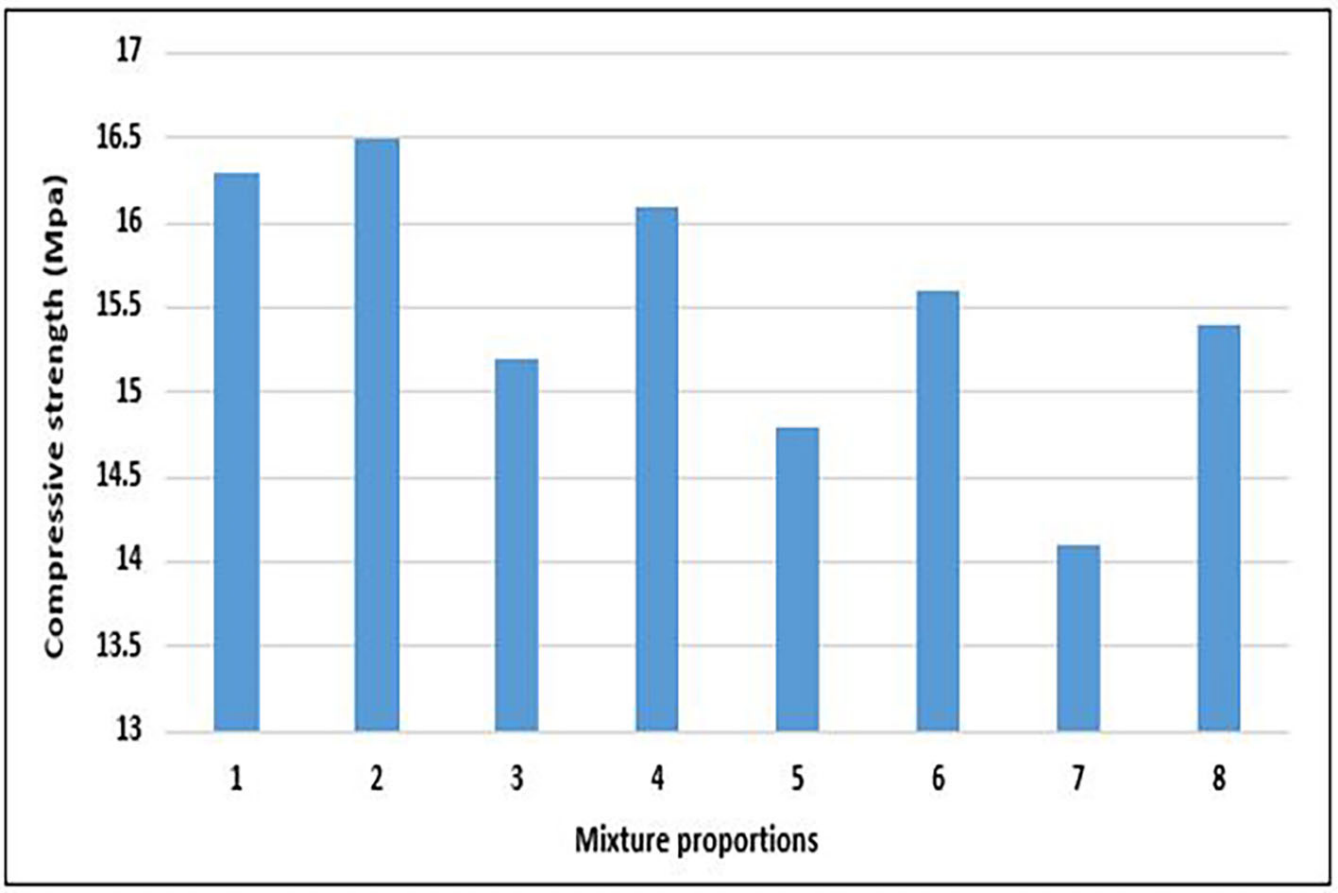
Compressive strength test results for mixture proportions.

The compressive strength is the primary quality of concrete that determines the extent of its application. This is a fundamental measure of the ability of a concrete specimen to resist direct axial stress. In many classical studies, the 28-day compressive strength is the most commonly used metric, regarded as a fundamental benchmark for the design, production, and application of concrete ( Chou & Pham, 2013). The compressive strengths of the PET concrete specimens were determined, and the results are shown in Figure 13.

The concrete specimens attained compressive strength of 14.1 to 16.5 MPa with an average is 15.5 MPa ( Figure 13). According to BS EN 206:2013+A2:2021 ( British Standards Institution, 2021), it falls under LC13 lightweight concrete (13–17.9 MPa). EN-1992-1-1:2004 ( European Committee for Standardization, 2004) stipulated LC13 as the lowest strength class for lightweight structural concrete. The majority of the mixture proportions, aside from mixes 5 and 7, attained a minimum compressive strength of 15 Mpa. This is an acceptable strength for structural applications following the CEB-FIP Model Code 90 ( Committee for The Model Code 1990, 1993) and the International Union of Laboratories and Experts in Construction Materials, Systems, and Structures (RILEM) ( Beushausen & Fernandez Luco, 2015). The compressive strength of the specimens from mixes 2, 1, and 4 falls marginally out of the ACI 213 R-14 ( American Concrete Institute, 2014) category of structural lightweight concrete. Mix 8 is considered by this study as the most environmentally friendly and sustainable mixture proportion because it attains an acceptable strength with the highest quantity of PET aggregates.

**Figure 14.**
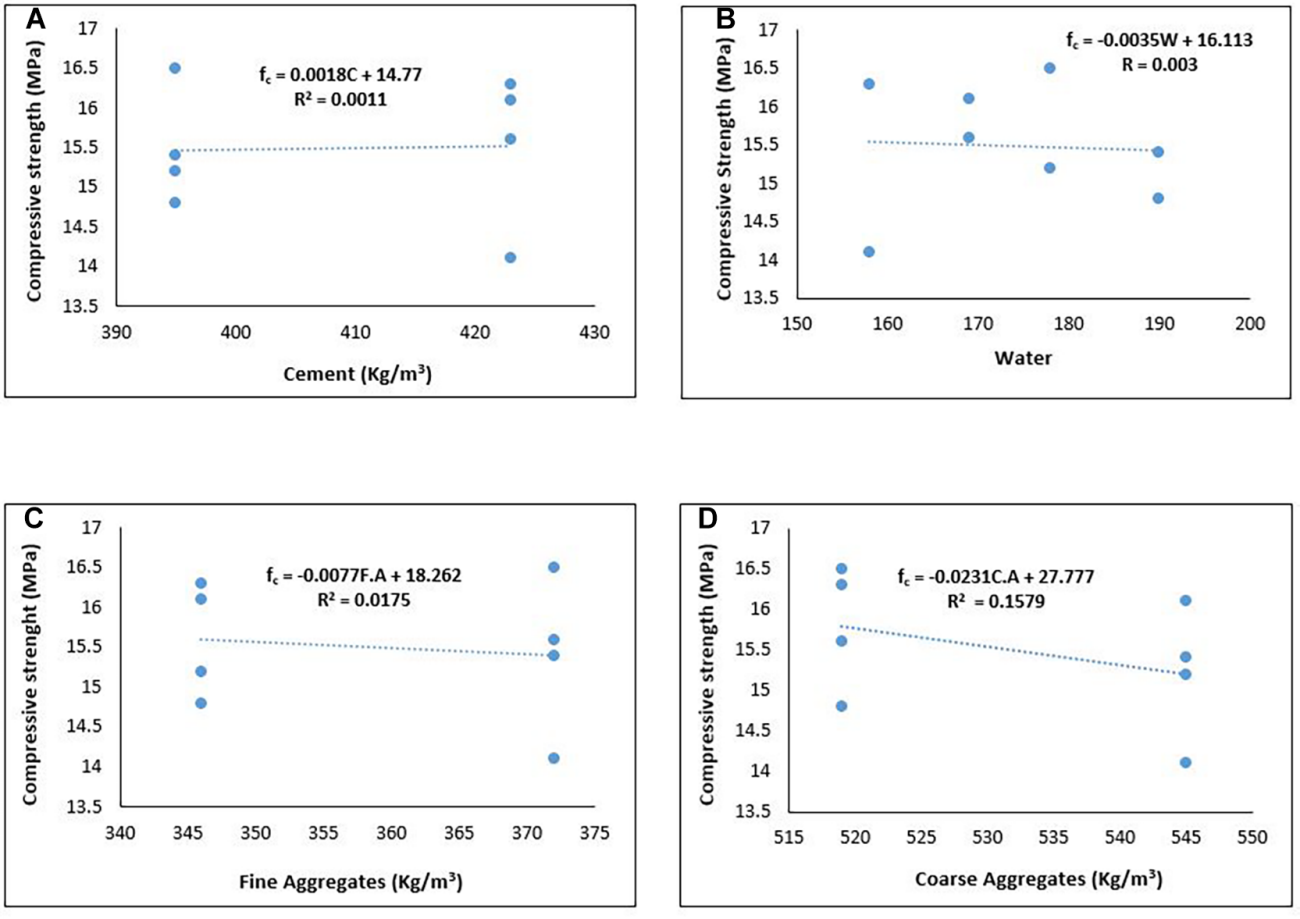
(a) Relationship between compressive strength and cement content. (b) Relationship between compressive strength and water. (c) Relationship between compressive strength and fine aggregates. (d) Relationship between compressive strength and coarse aggregates.

Figure 14 shows the relationship between the compressive strength and components of the PET lightweight aggregate concrete.

As shown in Figure 14, the compressive strength of PET lightweight aggregate concrete exhibits a positive relationship with cement content and a negative relationship with water, fine aggregates, and coarse aggregate content. Thus, the ideal mixture proportion for lightweight structural concrete comprising PET aggregates has a higher cement content than water, fine aggregates, and coarse aggregates. Figure 14a and 14b infers that a reduction in water/cement ratio will result in greater compressive strength. From Figure 14c and 14d, the negative impact of the coarse aggregates was more prominent than that of the fine aggregates. This implies that a reduction in the quantity of coarse aggregates and increase in the quantity of fine aggregates positively reflects on the compressive strength. This observation further suggests that it is more desirable to replace a portion of PET coarse aggregates with stronger lightweight or normal-weight aggregates to improve compressive strength. The strength of the aggregate and the interplay between the aggregate and cement paste are limiting factors in the compressive strength development of lightweight PET concrete ( Bogas & Gomes, 2013b; K. Thienel et al., 2020).

PET aggregates have been incorporated into concrete as fine or coarse aggregates, with significant results in terms of compressive strength ( C. K. A. Uche et al., 2023). Substituting fine aggregates with PET aggregates up to 50% yielded a compressive strength ranging from 21 MPa to 25 MPa ( El-Nadoury, 2022; Hanuseac et al., 2020; N. Hilal et al., 2022; Kangavar et al., 2022; Qaidi et al., 2023). Hanuseac et al. ( Hanuseac et al., 2020) substituted 70% and 90% of fine aggregates with PET aggregates, leading to concrete with compressive strengths of 20 MPa and 15 MPa, respectively. Replacing 100% of fine aggregates with PET aggregates yields concrete with a compressive strength of 16.3 MPa ( Bamigboye et al., 2021). Regarding coarse aggregates, a replacement level of 50% with PET aggregates, manufactured using procedures similar to this study by ( Bamigboye et al., 2022; Islam et al., 2016), yielded compressive strengths of 13 MPa and 20.5 MPa, respectively. Complete substitution at 100% level with aggregates of identical qualities yielded concrete with compressive strengths of 12 MPa and 17 MPa, respectively ( Bamigboye et al., 2022; Nursyamsi & Zebua, 2017). A similar value of 13MPa for compressive strength was obtained by ( C. Uche et al., 2022) after total substitution with PET aggregates. Replacement with fine PET aggregates demonstrated superior strength compared to coarse PET aggregates. This corroborates the conclusions of the study. The compressive strength values derived from this investigation with complete aggregate replacement are comparable to those from partial replacement, thereby reinforcing this method as environmentally sustainable.

Research on the compressive strength of lightweight concrete produced using different lightweight aggregates has been conducted. All lightweight concrete produced from shale, pumice and expanded clay, attained compressive strengths of 34.3, 31.4 and 31.3 MPa, respectively ( Nguyen et al., 2014). Thermally expanded clay (TEC) and calcined clay (CC) as coarse and fine aggregates were combined in all lightweight concrete, and a compressive strength between 23.72 and 30.23 MPa was obtained ( Palacios et al., 2020). Majhi et al. ( Majhi et al., 2021) used sintered fly ash as fine aggregates and fly ash cenosphere as coarse aggregates to produce lightweight structural concrete of 17.57 MPa compressive strength. Bottom ash aggregate was used to replace natural fine and coarse aggregates in lightweight aggregate concrete, resulting in a compressive strength of 23.3 to 41.3 MPa ( Kim et al., 2020). A compressive strength similar to the result of this study was obtained by ( Yaşar & Erdoǧan, 2008) for all lightweight concrete with pumice aggregates. Uche et. al. ( C. Uche et al., 2022); an earlier study recorded a maximum compressive strength of 12.56 MPa from all-lightweight concrete with PET aggregates.

### 4.4 Splitting tensile strength of concrete

**Figure 15.**
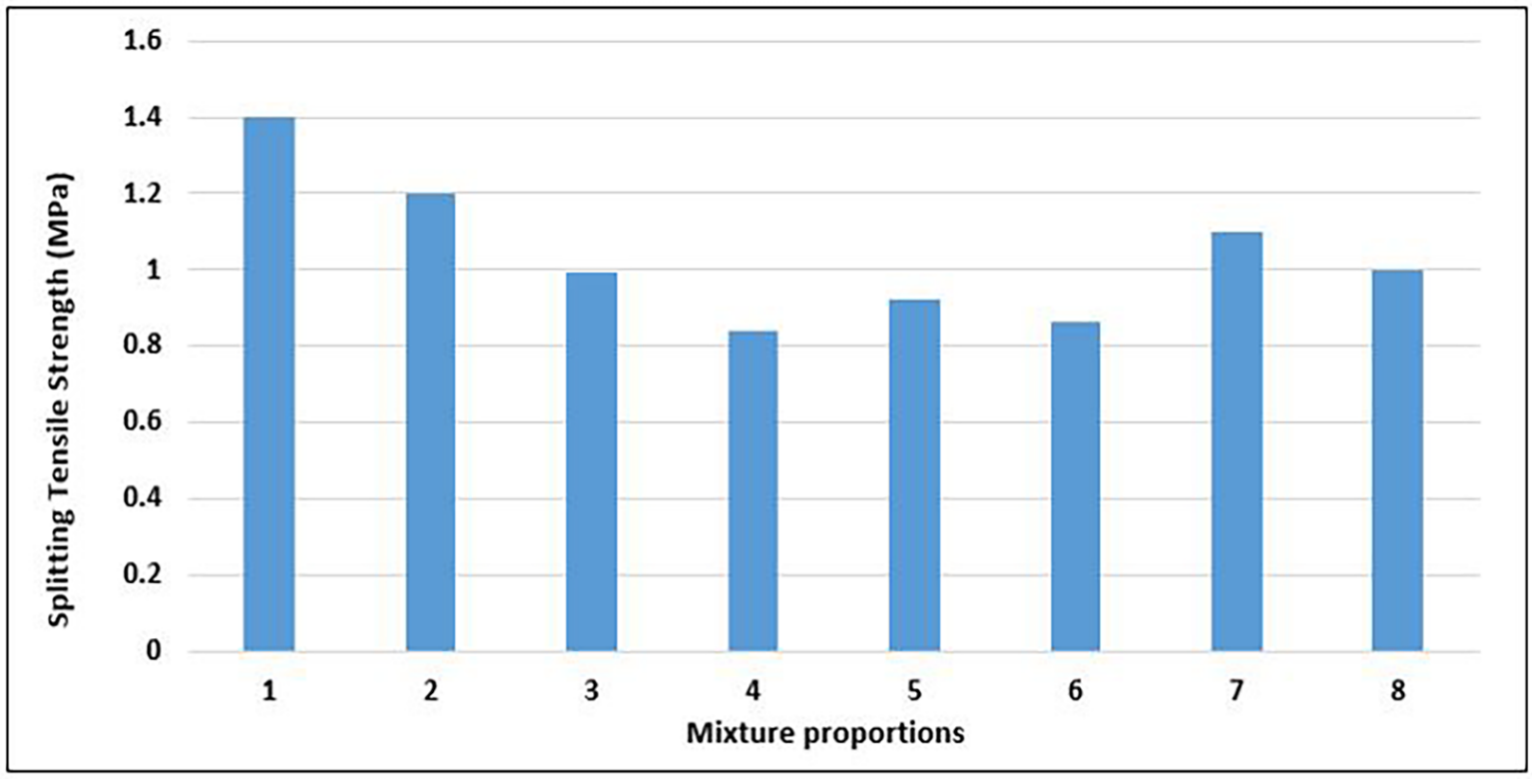
Splitting tensile strength test results for mixture proportions.

The splitting tensile strength of concrete is a fundamental property that significantly influences the degree and amount of cracking in structures. This is the capacity of a concrete specimen to overcome tensile stress. The splitting tensile strength is a crucial metric for evaluating the structural integrity and durability of concrete in a variety of construction applications, considering that concrete is brittle and weak in tension. The results of the splitting tensile strength test on the concrete specimens with different mixture proportions are displayed in Figure 15.

The splitting tensile strengths of the concrete specimens range from 0.84 to 1.4 MPa, with an average value of 1.04 MPa ( Figure 15). Mix 1 had the highest splitting tensile strength, whereas Mix 4 had the lowest splitting tensile strength of 40%. Mixes 1, 2,7 and marginally mix 8 attained values that satisfied the minimum splitting tensile strength of 1.1 MPa for structural concrete, as prescribed by CEB-FIP Model Code 90 ( Committee for The Model Code 1990, 1993). The value of Mix 1 exceeds 1.3Mpa, which is the minimum value for lightweight structures according to EN -1992-1-1:2024 ( European Committee for Standardization, 2004). Most of the mix proportions that attained a high splitting tensile strength had a higher amount of fine aggregates, while most of those with a low splitting tensile strength had a lower fine aggregate content. The angular nature and ductility of the PET aggregates created an interlocking mesh within the concrete matrix, which provided more resistance to the splitting forces. The failure mode of the concrete specimens was ductile, rather than brittle, drawing a comparison with the work of ( Qaidi et al., 2023).

The inclusion of PET aggregates in structural lightweight concrete produced varying splitting tensile strength results. Results of splitting tensile strength tests after 50% of fine aggregates have been replaced by PET aggregates are in the range of 2 MPa to 3.5 MPa ( Bamigboye et al., 2021; El-Nadoury, 2022; Kangavar et al., 2022; Qaidi et al., 2023). Similar to compressive strength, the inclusion of coarse PET aggregates led to lower splitting tensile strength of concrete compared to fine PET aggregate replacement. Bamigboye et. al., ( Bamigboye et al., 2022) recorded splitting tensile strength values of 1.3 MPa and 1.2 MPa with 50% and 100% substitution of natural coarse aggregates with PET coarse aggregates respectively. The maximum value of 1.4MPa obtained from the total replacement of natural aggregates with PET aggregates in this study is appreciable in comparison.

A splitting tensile strength of 1.52 MPa was obtained from structural lightweight concrete with sintered fly ash as fine aggregates and fly ash cenosphere as coarse aggregates ( Majhi et al., 2021). Cinder lightweight aggregate was used to replace 100% of the coarse aggregates in ( Sahoo et al., 2022). The resulting concrete exhibited a splitting tensile strength of 2.81MPa. All-lightweight concrete produced with pumice aggregates recorded splitting tensile strengths in the range of 2 to 2.4 MPa ( Parhizkar et al., 2012). Karthika et. al. ( Karthika et al., 2020) obtained a splitting tensile strength of 1.36 Mpa after replacing coarse aggregates with pumice lightweight aggregates. This result was close to the maximum splitting tensile strength observed in this study.

### 4.5 Water absorption of concrete

**Figure 16.**
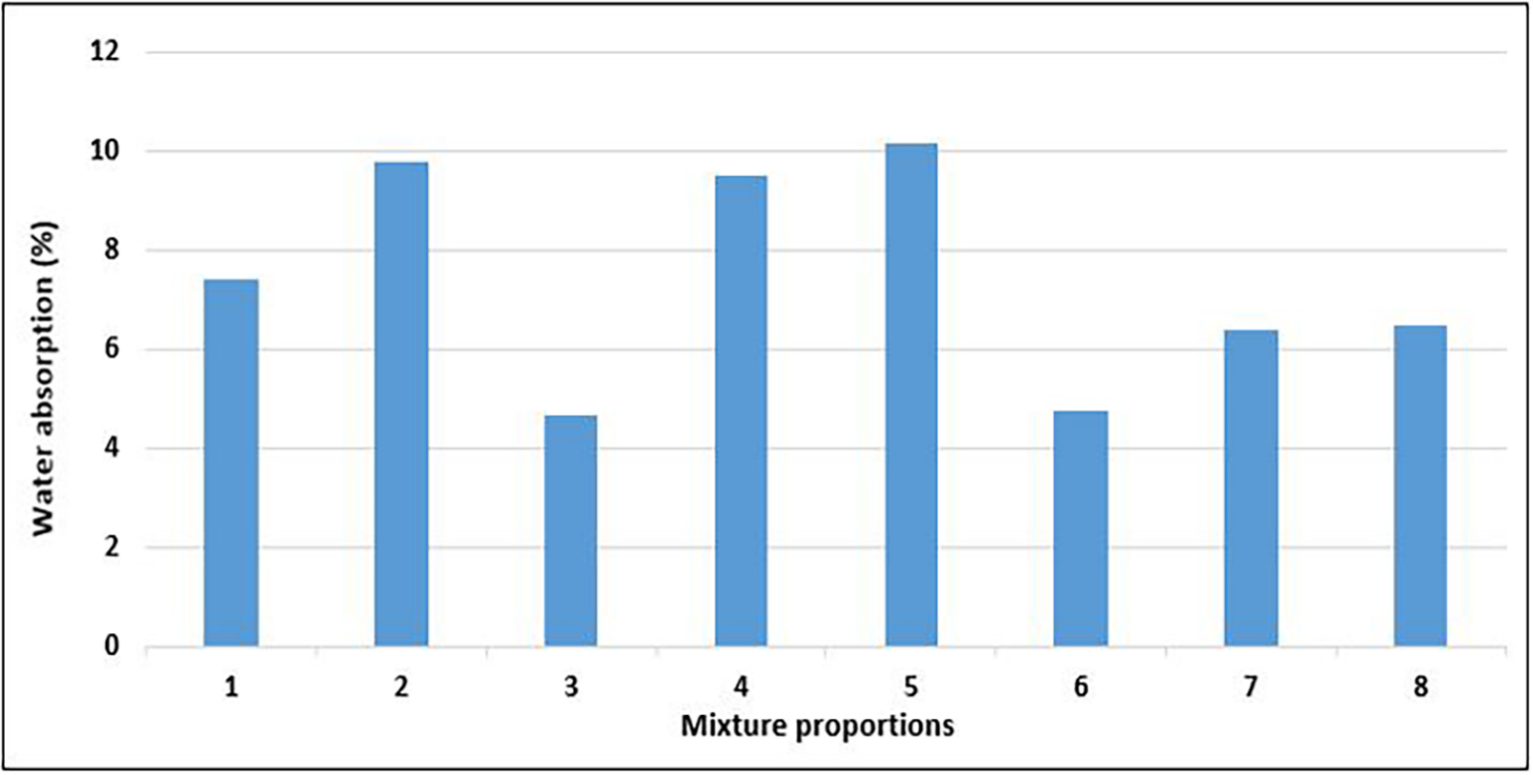
Water absorption test results for mixture proportions.

Water absorption is a common metric used to evaluate the durability of concrete under various environmental conditions ( Folagbade, 2016). Durability is an important consideration in the field of construction because it is the ability of a concrete structure to preserve its serviceability ( Czarnecki et al., 2020). Furthermore, the extent to which the microstructure of concrete permits the entry of a fluid indicates the durability of concrete. This entry of fluids can introduce molecules that degrade the stability of concrete through chemical reactions ( Mehta & Monteiro, 2006). Figure 16 shows the results of the water absorption tests for the PET concrete specimens.

As shown in Figure 16, the water absorption for the concrete specimens ranges from 4.66 to 10.16%. Mixes 3 and 6 had the lowest water absorption, whereas mixes 5 and 2 had the highest water absorption respectively. The amount of water absorbed by mix 3 was 54% less than that absorbed by mix 5. Following the CEB-FIB 192 ( CEB-FIB, 1989) concrete obtained from mixes 3 and 6 is average quality concrete, while those from the other mixture proportions are considered poor quality concrete. Mix 7 and 8 with the highest quantity of PET aggregates recorded water absorption below the average value of 7.4%. According to the requirements given in BS EN 12390-8:2019 ( British Standards Institution, 2019d), every concrete specimen that was evaluated satisfied the requirements for lightweight concrete when shielded from atmospheric influence (WA < 25%). All concrete specimens further satisfied the more rigorous criterion of < 20% water absorption for cases of unprotected concrete under atmospheric conditions. Neville ( Neville, 2011) considered a maximum water absorption of 10% as the benchmark for high-quality concrete. Mixes 5 and 2, which had the highest permeability, had a high water content, resulting in excess water, which led to the existence of many void spaces in the solid concrete matrix. The permeability of concrete is closely correlated with the properties of the internal structure of the cement paste and the degree of microcracks at the aggregate-cement boundary and within the cement paste ( De Schutter & Audenaert, 2004).

The effect of the inclusion of PET aggregates on the water absorption of concrete has been well documented. In the study of ( Dawood et al., 2021), a water absorption rate of 2.41% was observed for concrete samples with a 20% replacement of PET aggregates. A substitution of 50% PET fine aggregates by ( El-Nadoury, 2022; Qaidi et al., 2023) resulted in 5.7% and 7.8% water absorption respectively. The results of ( Al-Hadithi & Al-Ani, 2018) showed that as the concrete aged, the absorption values of all the specimens decreased continuously. Water absorption values obtained from this study with 100% PET aggregates compare relatively better than the values from the reference studies, with a minimum and average water absorption of 4.5% and 7.4%.

Structural lightweight concrete with various components and proportions was subjected to water-absorption tests. All-lightweight concrete using sintered fly ash and fly ash cenosphere as fine and coarse aggregates, respectively, exhibited a water absorption of 17.1% ( Majhi et al., 2021). Similarly ( Parhizkar et al., 2012) produced lightweight concretes using pumice aggregate and observed water absorption in the range of 10.2 to 11.22%. An even higher value range of 14 to 22% was obtained by ( Gündüz & Uǧur, 2005) for all-lightweight concrete with pumice aggregates. The concrete specimens from this study exhibited better results than those mentioned above. The use of lightweight PET aggregates results in more durable concrete compared to lightweight aggregates from natural or artificial sources. Water absorption ranges from 5 to 25% in lightweight structural concrete ( Przychodzien & Katzer, 2021).

## 5. Empirical relationships between properties of structural lightweight concrete

Noteworthy empirical relationships between various properties were identified, and regression models were developed from experimental data to describe these relationships.

### 5.1 Relationship between workability and fresh density

**Figure 17.**
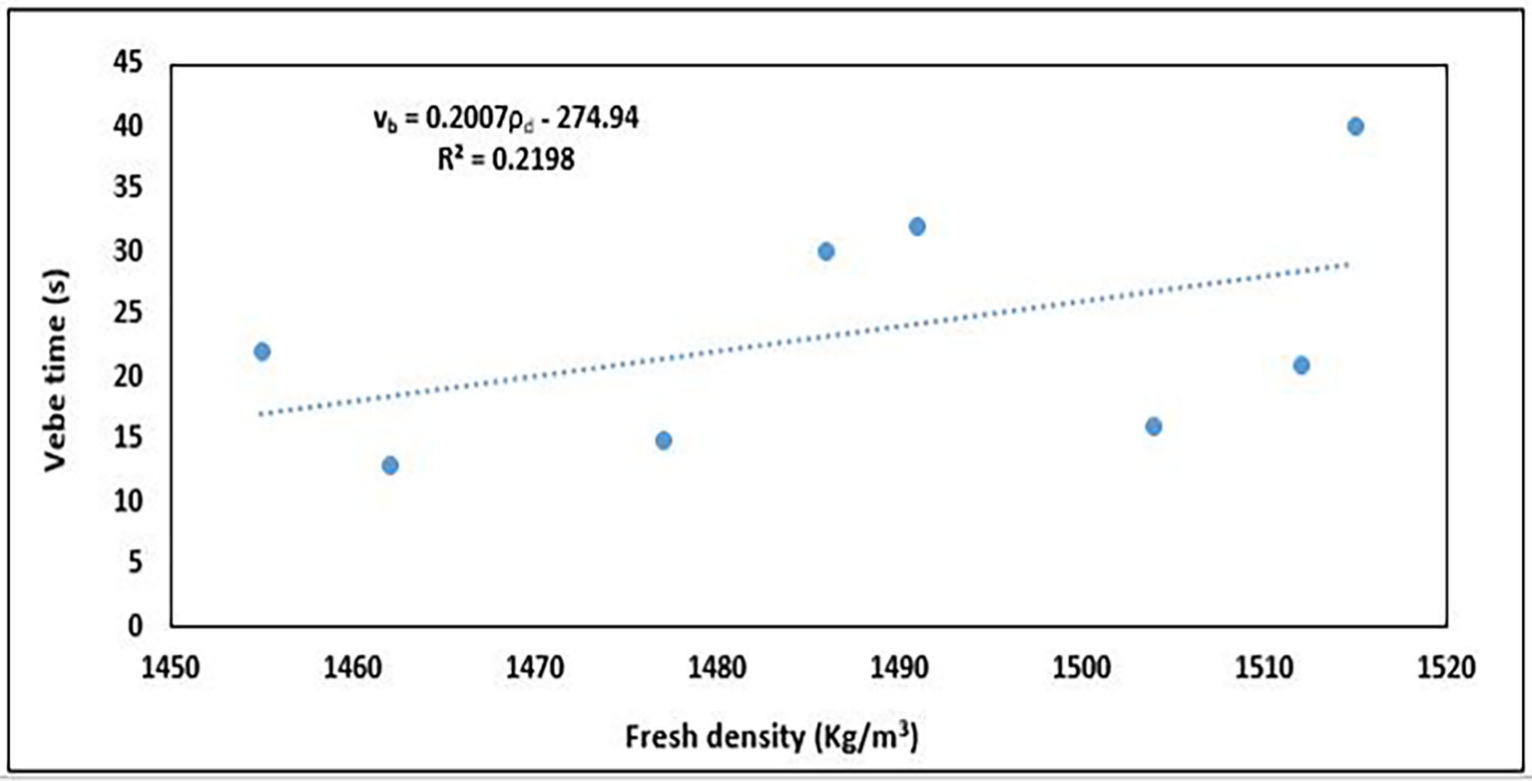
Relationship between workability and fresh density.

A lower fresh density owing to the increased air content and reduced aggregate packing efficiency typically results in higher workability. Conversely, lower workability often corresponds to a higher fresh density, as the mix is more compact and contains less entrapped air. The relationship between the fresh concrete properties, workability, and density was determined, as shown in Figure 17.

The relationship in Figure 17 is best described by a linear equation thus:

$$v_{b}=0.2007\rho_{d}-274.94$$ (10)

where $v_{b}$ is the workability in vebe time (s) and $\rho_{d}$ is the fresh density of the concrete (Kg/m ^3^).

The results revealed a positive correlation with a slope of ≅0.2 , indicating that as fresh density increases, the vebe time increases with attendant reduction in workability. The relationship between both properties was weak, as shown by the coefficient of determination R ^2^ value of 0.2198 indicating that 21.98% of the variation in vebe time can be explained by changes in fresh density. The less-dense concrete mixes collapse more easily than the denser mixes, which provides greater resistance to the vibration of the vebe machine owing to their more compact nature.

### 5.2 Relationship between compressive strength and workability

**Figure 18.**
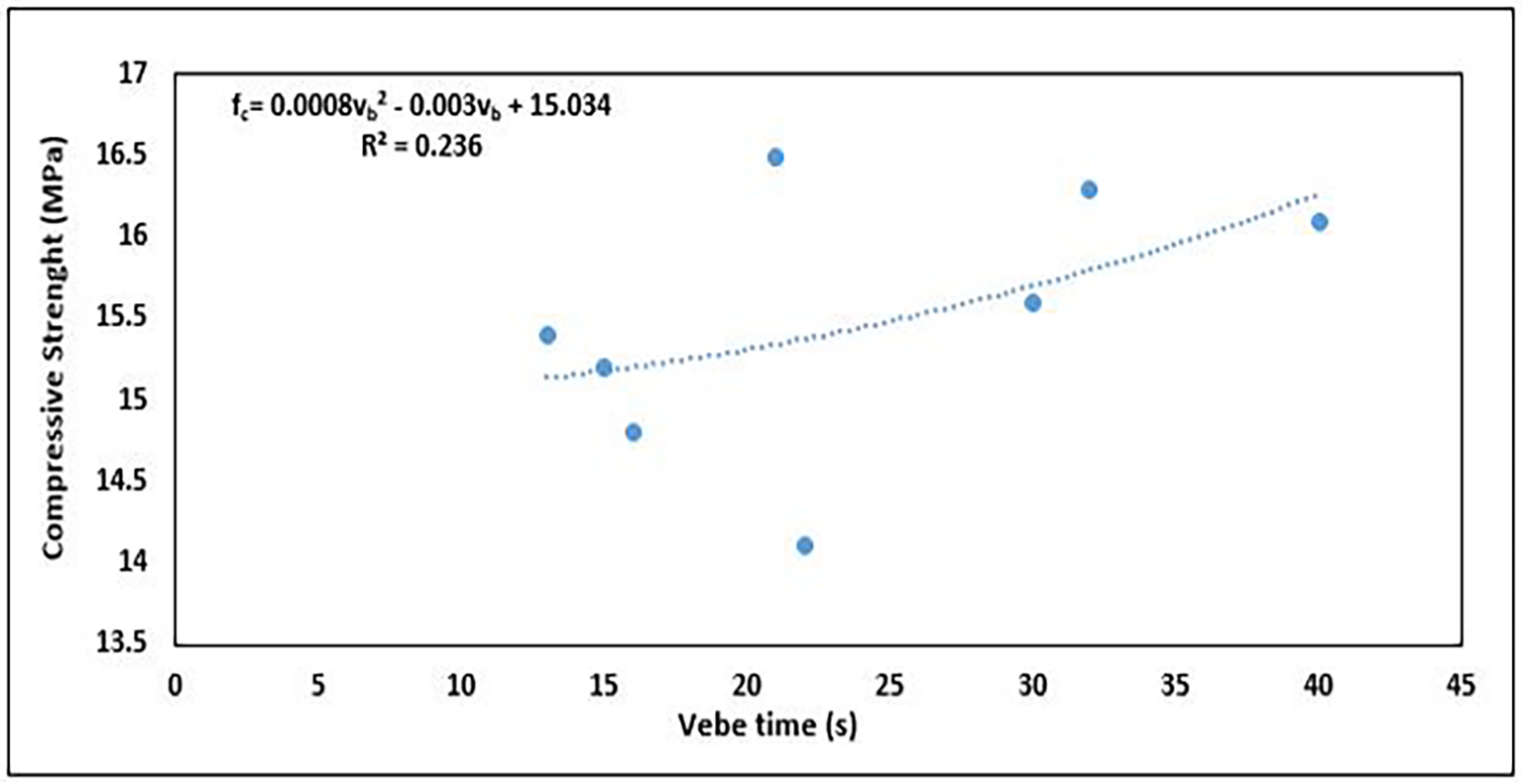
Relationship between workability and compressive strength.

Generally, as the workability increases, the compressive strength tends to decrease in most cases because of the higher water content. This is because an increase in the water content leads to a higher water-cement ratio, thereby reducing the density and strength of the hardened concrete. Conversely, low workability (insufficient water) can lead to poor compaction and air voids, which also reduces the compressive strength. Figure 18 shows the correlation between workability and compressive strength.

The relationship is of a 2 ^nd^-order polynomial ( Figure 18), and the equation is

$$f_{c}=0.0008v_{b}^{2}-0.003v_{b}+15.034$$ (11)

where $v_{b}$ is the workability in vebe time (s) and $f_{c}$ is the compressive strength (MPa).

These properties have a weak positive relationship, as seen in the R ^2^ value of 0.236, with only 23.6% of the variability in compressive strength being explained by the quadratic model of vebe time. The positive trend of the relationship agrees with the general assumption of greater strength and lower workability. The ideal mix proportion strikes a balance where the concrete has sufficient workability for ease of placement while maintaining adequate compressive strength.

### 5.3 Interaction of compressive strength and dry density

**Figure 19.**
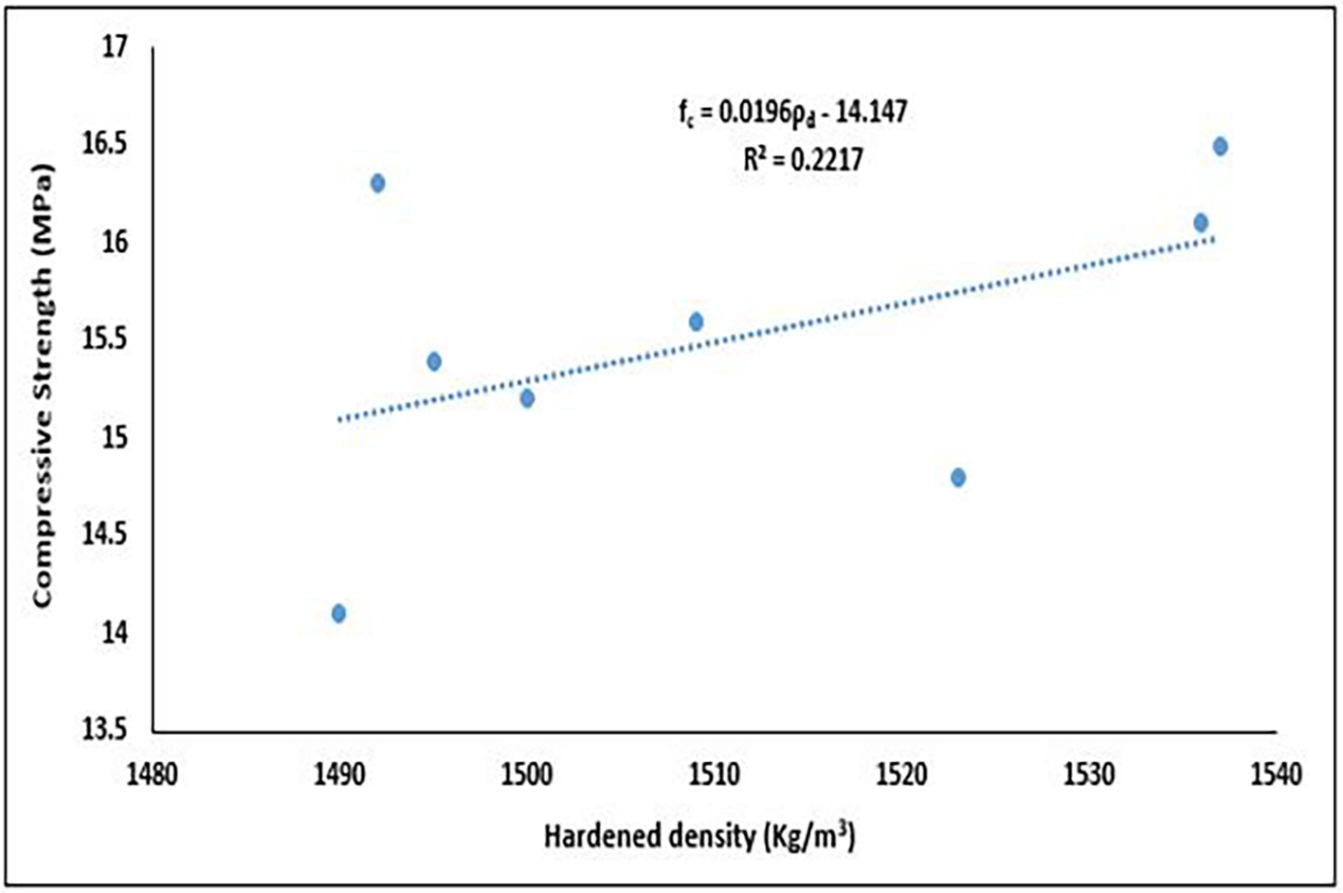
Relationship between dry density and compressive strength.

The interactions between the dry density and compressive strength of concrete are crucial for determining the quality, durability, and suitability of the material for various structural applications. Figure 19 illustrates the interaction between the compressive strength and hardened density of the concrete specimens.

The best fit for this relationship in Figure 19 is linear viz:

$$f_{c}=0.00196\rho_{d}-14.147$$ (12)

where, $\;\rho_{d}$ is the Dry Density (kg/m ^3^), $f_{c}$ is the Compressive Strength (MPa).

The results revealed a positive correlation with a slope of 0.0196, indicating that as dry density increases, compressive strength tends to rise. The correlation coefficient (r = 0.471) suggests a moderate linear association influenced in part by two outlier points. The coefficient of determination (R ^2^ = 0.2217) indicates that approximately 22.17% of the variation in compressive strength can be explained by changes in dry density. The observed trend aligns with literature, which typically reports a positive correlation between these properties. As dry density increases, pore spaces in the concrete matrix are progressively filled by hydration products, resulting in reduced porosity and a denser microstructure ( Chen, 2023; Ge et al., 2023). Consequently, mixtures with higher dry densities (e.g., Mixes 2, 4, and 5) are more likely to achieve structural-grade compressive strength. This relationship is particularly relevant in PET-based concrete, where the hydrophobic and lightweight nature of PET aggregates can compromise bonding and strength unless countered by optimized mixture proportions ( Orie, 2023; C. K. A. Uche et al., 2023). Overall, the correlation between density and strength serves as a valuable indicator of concrete quality and structural adequacy. Significant deviations from this expected trend may suggest deficiencies in mix design, compaction, or curing practices.

Another significant expression of this relationship is concrete structural efficiency. This is the ratio of compressive strength to dry density. Table 8 shows the structural efficiency of the PET concrete.

**Table 8.** Structural efficiency of PET lightweight concrete.

Mix	Hardened Density (Kg/m ^3^)	Compressive Strength (MPa)	Structural Efficiency kPa·m ^3^/kg
1	1492	16.3	10.9
2	1537	16.5	10.7
3	1500	15.2	10.1
4	1536	16.1	10.5
5	1523	14.8	9.7
6	1509	15.6	10.3
7	1490	14.1	9.5
8	1495	15.4	10.3
Average	1510	15.5	10.3

From Table 8, mixes 1 and 2 are the most efficient mix proportions compared with mixes 5 and 7. These values are larger than the lowest possible values of 6.5 and 8.9 according to EN 1992-1-1:2004 ( European Committee for Standardization, 2004) and ACI 213 – R14 ( American Concrete Institute, 2014). Lightweight concrete has a typically high strength-to-weight ratio because it achieves reasonable compressive strength while significantly reducing the dead load. This makes it suitable for high-rise buildings, bridge decks, and precast panels, where weight minimization is crucial.

### 5.4 Relationship between dry density and water absorption

**Figure 20.**
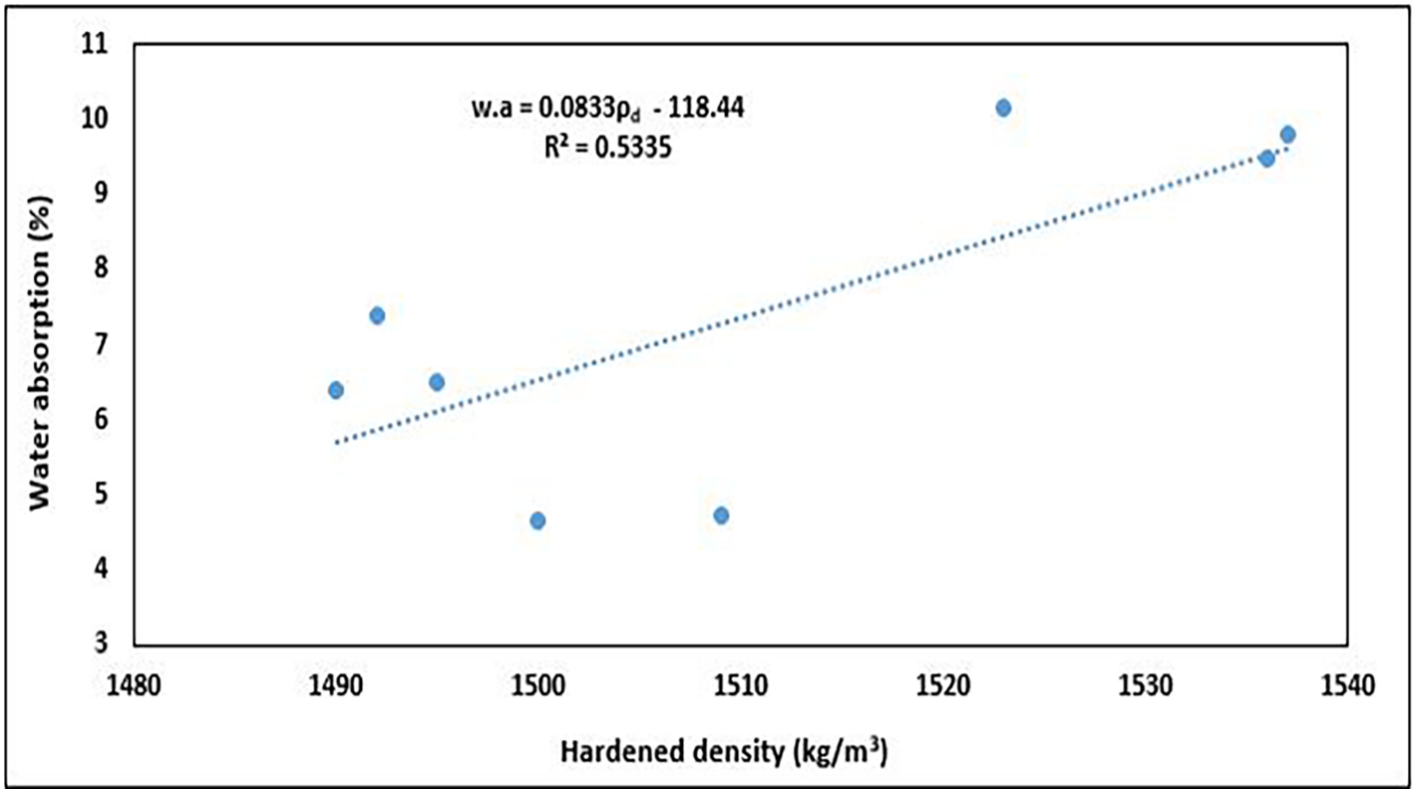
The relationship between dry density and water absorption.

The relationship between dry density and water absorption of concrete is essential for evaluating its durability and long-term serviceability. Figure 20 shows the relationship between the hardened density and water absorption of PET concrete specimens.

The water absorption increased with increasing density ( Figure 20). The equation used to describe this observation is:

$$w.a=0.0833\rho_{d}-118.44$$ (13)

where w.a is the water absorption and $\rho_{d}\;$ is the hardened density of the concrete specimens.

The relationship was positive, with an R ^2^ of 0.5335, signifying a good interaction between the properties, which suggests that about 53.35% of the variation in water absorption can be explained by the variation in dry density. This positive tendency is dissimilar to that of other authors, who observed a negative trend of reduced water absorption with an increase in hardened density in structural lightweight concrete ( Akinwumi et al., 2014; Hasan et al., 2021; Majhi et al., 2021; Shafigh et al., 2014). This is because a denser concrete matrix has fewer and smaller voids, thereby reducing the pathways for water to penetrate the material. This relationship helps engineers design concrete with enhanced durability and longevity, particularly in environments that are exposed to moisture and aggressive chemicals. Concrete with low water absorption is more resistant to freeze-thaw cycles, chemical attacks, and other forms of environmental degradation, making it more durable in the long term.

### 5.5 Relationship between splitting tensile strength and compressive strength

**Figure 21.**
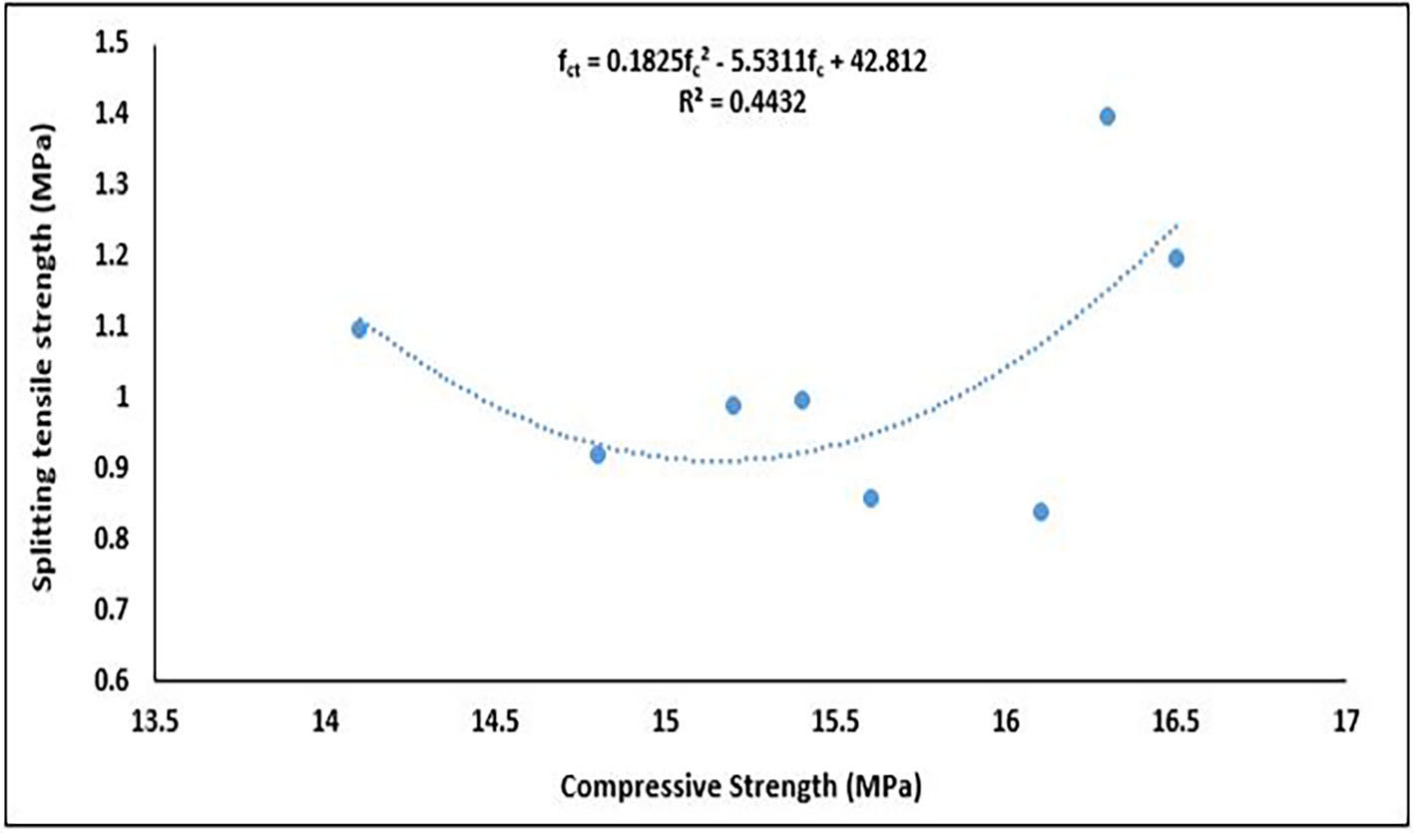
Relationship between compressive strength and splitting tensile strength.

Tensile and compressive strengths can only have an empirical relationship, as the factors affecting both properties are dissimilar ( Almeshal, I. et al., 2020). The splitting tensile strength is a function of the compressive strength; therefore, it is necessary to determine the relationship between the two strength properties. Typically, in concrete design, the compressive strength is first established, followed by an estimation of the tensile strength using an empirical relationship ( Tennis et al., 2004). A more robust structural design can be achieved with greater knowledge of their interactions. This relationship is illustrated in Figure 21.

The splitting tensile strength had a polynomial relationship with the compressive strength ( Figure 21). This relationship is mathematically represented as:

$$f_{\mathrm{ct}}=0.1825{f_{c}}^{2}-5.5311f_{c}+42.812$$ (14)

where, $f_{c}$ is compressive strength, and $f_{\mathrm{ct}}$ is splitting tensile strength.

The relationship between these properties was fair, with a coefficient of determination R ^2^ = 0.4432, meaning 44.32% of the variation in tensile strength is explained by the variation in compressive strength through this quadratic model. A similar 2 ^nd^-order polynomial relationship was obtained in ( Shiuly et al., 2022; Tayeh et al., 2021). The quadratic regression suggests that tensile strength is not consistently proportional to compressive strength, likely due to the influence of PET content on bond structure, crack propagation and matrix cohesion. This relationship is essential for structural design because the tensile strength is much lower than the compressive strength. Empirical formulas based on this relationship are used in concrete design to estimate the tensile strength from the compressive strength, particularly when direct tensile testing is difficult. These models are crucial for the design of durable and crack-resistant concrete structures.

An empirical equation for splitting tensile strength based on the compressive strength of structural lightweight concrete was found in EN1992-1-1:2004 ( European Committee for Standardization, 2004). The equation used is as follows:

flctm=(0.3∗flck23)∗(0.4+0.6ρ2200) (15)

where $f_{\text{lctm}}$ is the mean splitting tensile strength for lightweight concrete, $f_{\mathrm{lck}}$ is the mean cylinder compressive strength of lightweight concrete, and ρ is the upper limit of the oven-dry density for the relevant density class (in this case = 1600Kg/m ^3^). In this procedure, the cube strength for lightweight concrete was converted to cylinder strength using a factor of 0.9.

**Figure 22.**
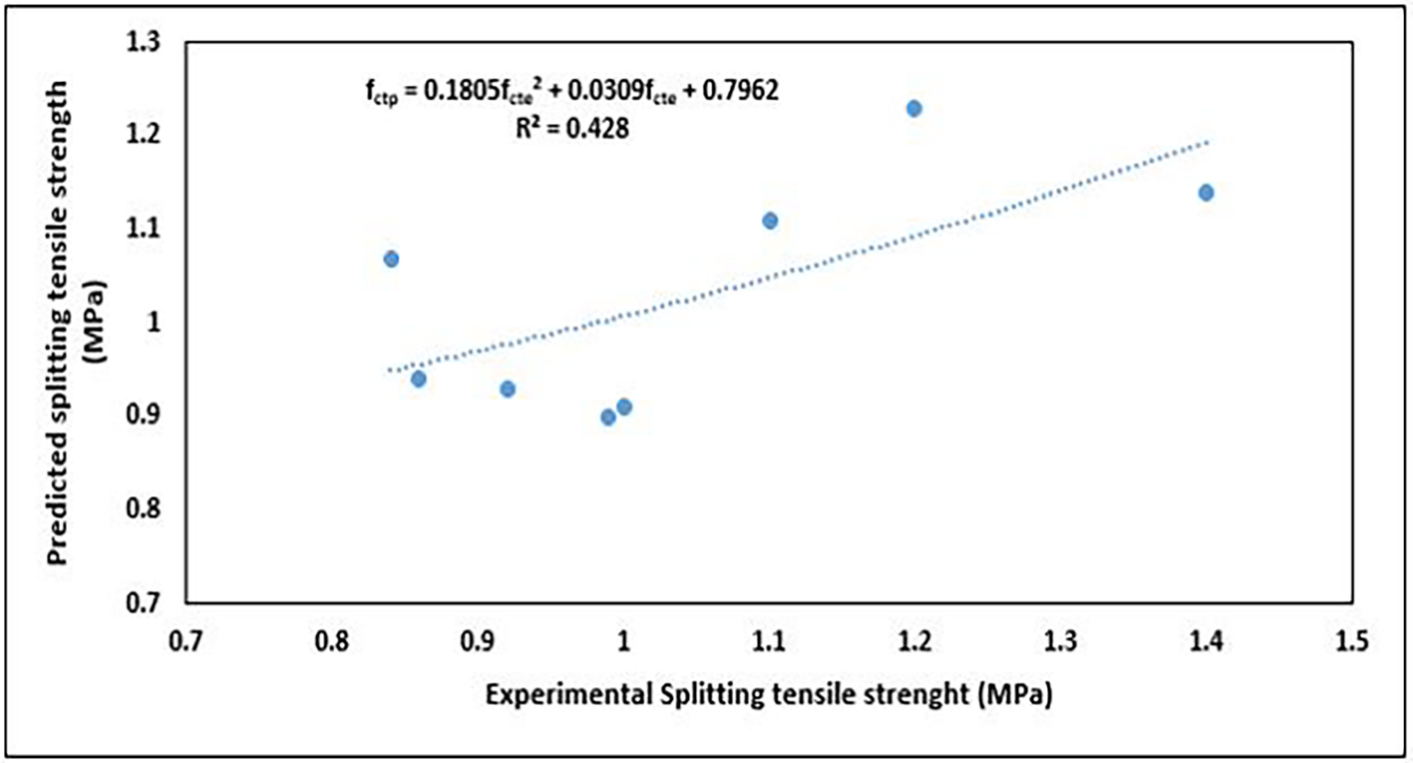
Relationship between predicted and experimental splitting tensile strength values.

**Figure 23.**
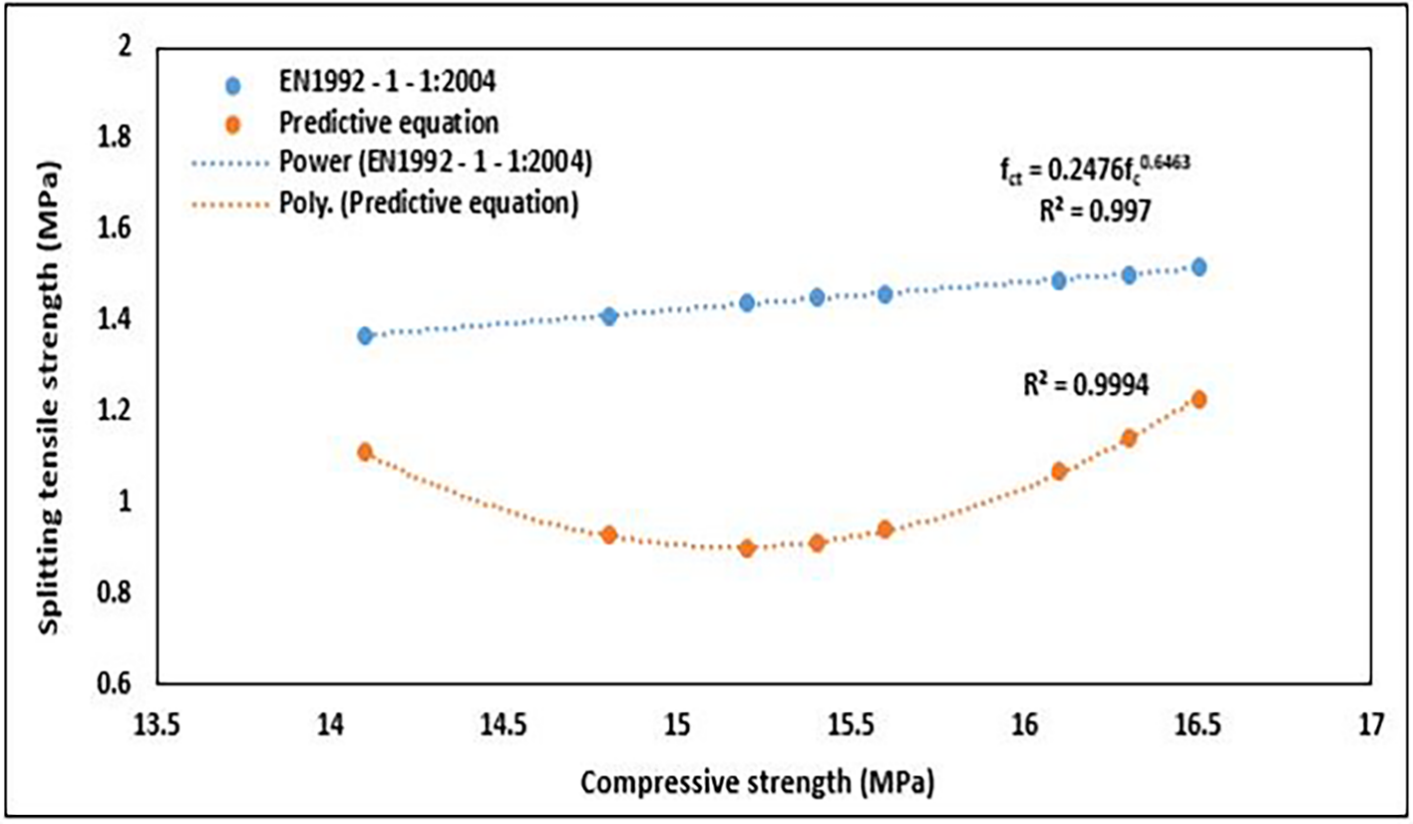
Comparison of splitting tensile strength values.

The splitting tensile strength values obtained from the predictive equation and EN1992-1-1:2004 ( European Committee for Standardization, 2004) are presented in Table 9. The predicted values were compared with the values obtained from experiments and EN1992-1-1:2004 ( European Committee for Standardization, 2004). These relationships are shown in Figures 22 and 23, respectively.

**Table 9.** Splitting tensile strength values from the predictive equation and EN1992-1-1:2004.

Compressive strength (MPa)	Splitting tensile strength (MPa)		
	Experimental value	Predictive equation $f_{\mathrm{ct}}=0.1825{f_{c}}^{2}-5.5311f_{c}+42.812$	EN1992-1-1:2004 flctm=(0.3∗flck23)∗(0.4+0.6ρ2200)
16.3	1.4	1.14	1.5
16.5	1.2	1.23	1.52
15.2	0.99	0.9	1.44
16.1	0.84	1.07	1.49
14.8	0.92	0.93	1.41
15.6	0.86	0.94	1.46
14.1	1.1	1.11	1.37
15.4	1	0.91	1.45

The relationship described by the 2 ^nd^-order polynomial ( Figure 22) is as follows:

$$f_{\mathrm{ctp}}=0.1805f_{\mathrm{cte}}^{2}+0.0309f_{\mathrm{cte}}+0.7962$$ (16)

where $f_{\mathrm{ctp}}$ is the predicted splitting tensile strength (MPa) and $f_{\mathrm{cte}}$ is the experimental splitting tensile strength (MPa).

This is a fairly good relationship because the predicted values mostly approximate the experimental values, except for a few outlier points.

The predictive equation for splitting tensile strength derived from the experimental values underperformed compared to the equation EN -1992-1-1:2024 ( European Committee for Standardization, 2004) which predicted higher values of splitting tensile strength ( Figure 23). This observation can be justified because the tensile strength of structural lightweight concrete depends on the tensile strength of the coarse aggregate and mortar phases, in addition to the security of their bonds. Conventionally, tensile strength is considered a function of compressive strength; however, it is well recognized that this is merely a preliminary estimate that does not account for surface features, moisture content, or distribution of the aggregate particle strength of concrete ( Holm & Ries, 2007).

The splitting tensile strength values from the equation EN -1992-1-1:2024 ( European Committee for Standardization, 2004) were related to the compressive strength by a power function; which is typical for the correlation of compressive strength and splitting tensile strength. The equation used is as follows:

$$f_{\mathrm{ct}}=0.2476f_{c}^{0.6463}$$ (17)

where $f_{c}$ is the compressive strength, and $f_{\mathrm{ct}}$ is the splitting tensile strength.

Error analysis was performed using the root-mean-square error (RMSE) to determine the most accurate predictive equation.

$$\text{RMSE}=\sqrt{\frac{1}{n}\sum_{i=1}^{n}{\left(E_{i}-P_{i}\right)}^{2}}$$ (18)

where, $E_{i}$ = Experimental values, and $P_{i}$ = Predicted values.

The results of this error analysis are seen in Table 10.

**Table 10.** Results of error analysis.

	Predictive equation $f_{\mathrm{ct}}=0.1825{f_{c}}^{2}-5.5311f_{c}+42.812$	EN1992-1-1:2004 flctm=(0.3∗flck23)∗(0.4+0.6ρ2200)
RMSE	0.389	0.449

From the error analysis results, the predictive equation had a lower RMSE value than the equation from EN -1992-1-1:2024 ( European Committee for Standardization, 2004). Hence, the predictive equation from this study is more accurate for predicting the splitting tensile strength from the compressive strength of PET structural lightweight concrete.

### 5.6 Water absorption and compressive strength relationship

**Figure 24.**
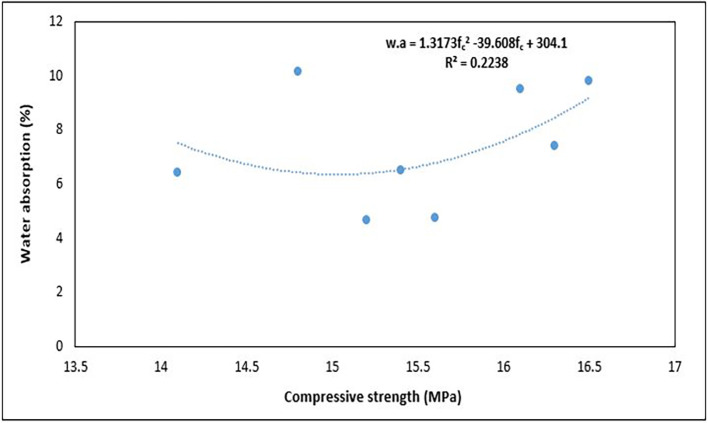
Relationship between water absorption and compressive strength.

The water absorption and compressive strength relationship in materials such as concrete or mortar is significant because it affects the durability and structural integrity of the material. Figure 24 shows the relationship between the water absorption and compressive strength for PET lightweight concrete.

The relationship between water absorption and compressive strength of PET concrete is shown in Figure 24. The relationship is described by a 2 ^nd^-degree polynomial equation as follows:

$$w.a=1.3173f_{c}^{2}-39.608f_{c}+304.1$$ (19)

where w.a is the water absorption (%) and f _c_ is the compressive strength (MPa).

The graph shows a weak positive trend with a coefficient of determination R ^2^ = 0.2238, indicating that only 22.38% of the variation in water absorption can be attributed to the compressive strength. The quadratic regression indicates that water absorption is not consistently proportional to compressive strength. This variability could be due to the low water/cement ratio and high cement content, leading to early microcracking. In addition, the hydrophobicity of PET aggregates has a negative influence on matrix integrity. The observed positive relationship represents a significant departure from the commonly observed trend of a negative relationship between water absorption and compressive strength ( El Ouni et al., 2022; S. P. Zhang & Zong, 2014). This relationship serves as a practical measure for assessing material quality, as increased water absorption raises the porosity of the material, weakening its internal structure and reducing its capacity to resist compressive forces. Lower water absorption suggests better structural integrity and resistance to environmental stressors ( Kumar et al., 2020; Razak et al., 2020).

## 6. Implications of regression models

The regression models yielded low coefficients of determination, reflecting weak linear or quadratic correlations among the investigated parameters. This is primarily attributable to the small sample size, which limits statistical power and increases sensitivity to outliers or anomalous results. Thus, the regression line is less stable and more influenced by individual data points. This limitation inherently restricts the achievable R ^2^ values, especially in complex behaviours like concrete properties.

Concrete properties are influenced by a multitude of factors: characteristics of the aggregates, water-cement ratio, admixtures, curing conditions, particle packing, etc. Additionally, mixes with only slight variations or uncontrolled variables—particularly the incorporation of plastic waste—will increase the scatter of data points. The heterogeneity in particle morphology and stiffness affects the concrete properties in non-uniform ways.

Furthermore, the relationships between the concrete properties are governed by complex, multivariate interactions. Simple bivariate models are insufficient to fully capture these dependencies, which likely involve synergistic effects from water–binder ratio, compaction efficiency, particle packing, and plastic content. Complex material behaviours may require more nuanced or multivariate models than simple linear or quadratic regressions.

The derived regression models are more indicative than predictive, with moderate explanatory power. They are useful in illustrating general trends and supporting preliminary conclusions.

## 7. Conclusion

This study developed mix designs focused on using polyethylene terephthalate (PET) aggregates as a full replacement for lightweight aggregates in structural lightweight concrete (SLWC). The research systematically evaluated the fresh and hardened properties, including density, workability, compressive strength, splitting tensile strength, and water absorption using standard test procedures while establishing empirical relationships between performance indices.

Conclusions drawn from this research are as follows:

1. Fresh and dry densities for all mixtures fell within the D1.6 category (1400–1600 kg/m ^3^), aligning with standard classifications for structural lightweight concrete and reflecting adequate compaction and matrix-aggregate bonding.
2. Workability (Vebe time: 13–40 s) remained low across all mixes despite superplasticiser use, attributed to PET’s hydrophobicity and angularity. The marginal influence of admixtures underscores the need for alternative workability-enhancing strategies (e.g., VMAs).
3. All mix designs achieved compressive strengths within the LC13 strength class (14.1–16.5 MPa), suitable for structural lightweight concrete, with mixes 2 and 4 showing peak strengths. Mix 8 achieved a balance of sustainability and strength, by achieving acceptable strength with the highest content of PET aggregates, thus making it the most environmentally friendly option.
4. The splitting tensile strength ranged from 0.84 to 1.4 MPa, with mixes 1, 2, 7, and 8 satisfying or exceeding the 1.1 MPa minimum structural requirement, indicating viable resistance to tensile stresses.
5. Water absorption values (4.66–10.16%) remained below the 20% threshold for unprotected lightweight concrete, indicating good durability performance even at high PET content levels.
6. Statistically significant but moderate correlations were established between the concrete properties, which were indicative of concrete behaviour and useful for illustrating general trends
7. Structural efficiency values (9.5–10.9 kPa·m ^3^/kg) confirm that PET-based lightweight concrete achieves favorable strength-to-weight ratios suitable for load-bearing applications.

The study validates PET aggregates as technically viable and environmentally beneficial materials in structural lightweight concrete, bridging the gap between waste valorization and structural performance.

## 8. Recommendations

The following are recommendations for future studies to enhance the understanding of structural lightweight concrete with chemically treated PET aggregates:

1. Evaluation of Additional Properties: Investigate key performance indices such as flexural strength, modulus of elasticity, thermal conductivity, chloride ion penetration and carbonation depth.
2. Microstructural Characterization: Conduct detailed microstructural analysis using Field Emission Scanning Electron Microscopy (SEM), X-ray Diffraction (XRD), and Energy Dispersive X-ray Spectroscopy (EDX) to examine the mineralogical, morphological, and chemical changes at the Interfacial Transition Zone (ITZ). These techniques will provide a deeper understanding of how the treatment with Ca (ClO) _2_ alters the hydration behavior, matrix interaction, and overall durability of PET-based structural lightweight concrete. In addition, particular attention is required in relation to the counterintuitive positive correlation between water absorption and dry density/compressive strength.
3. Sensitivity analysis: Future work should include DOE-based sensitivity analysis to isolate and rank the influence of individual and interacting parameters on the measured response.
4. Advanced Modelling Techniques: Employ multivariate regression or machine learning approaches with expanded datasets to better capture the interdependent factors influencing the behavior of PET-based lightweight concrete.
5. Sustainability Assessment: Quantify CO _2_ emissions for the various mix designs to identify the most environmentally sustainable formulation and minimize carbon footprint.
6. Optimisation of Mix Design: Develop an optimal mix design that maximizes the beneficial properties identified in this study, ensuring both structural efficiency and environmental sustainability.

The mix designs from this study can be adopted for the production of lightweight structures to encourage sustainable construction practices, reduce the impact of plastic pollution, conserve natural aggregate deposits and increase the structural efficiency of concrete members.

## Clinical trial number

Clinical trial number: Not applicable.

## Ethics statement

Ethics declaration: Not applicable.

## Consent to publish

Consent to Publish declaration: Not applicable.

## Consent to participate

Consent to Participate declaration: Not applicable.

## Data Availability

Figshare: Design and Evaluation of Sustainable Structural Lightweight Concrete Using Recycled PET as Aggregates. https://doi.org/10.6084/m9.figshare.30016696, Uche, Chikadibia (2025). The project contains the following underlying data: Data.csv. (PET_Lightweight_Concrete_Experimental_Data) Data are available under the terms of the Creative Commons license (CCO). Figshare: PET_SLWC_Concrete_Test_Results. https://doi.org/10.6084/m9.figshare.31076041, Uche, Chikadibia (2026). This project contains the following extended data: PET_SLWC_Concrete_Test_Results.csv Data are available under the terms of the Creative Commons license (CC0).
