# Supplementary material for: Design and Evaluation of Sustainable Structural Lightweight Concrete Using Recycled Pet as Aggregates
Source: F1000Res. 2026 Feb 14;14:922. Originally published 2025 Sep 15. [Version 2] doi: 10.12688/f1000research.168557.2 (PMC13182788; doi:10.12688/f1000research.168557.2)
Supplement: Peer review history

# Reviewer report: Reviewer response for version 2

**DOI:** 10.5256/f1000research.194690.r466639

**Published:** 2026-04-11

**Authors:** Adrián Bórquez-Mendivil

**Affiliations:** 1 Universidad Autonoma de Sinaloa, Culiacán, Sinaloa, Mexico

This article provides a very robust study on the physical and mechanical responses of the total replacement of conventional coarse aggregate by aggregates made from PET waste in the production of hydraulic concrete. However, having read and reviewed this document, I believe significant changes are needed for it to be considered acceptable according to established scientific standards in the community. These changes primarily concern the structure of the introduction, critical details in the methodology, clarity in the discussion of results, and highlighting the novelty of the work in both the introduction and the conclusions described below.

**Comment 1. Introduction: **The introduction contains useful information, but it lacks a coherent structure around the actual scope of the study and currently mixes several topics. The narrative jumps from PET waste to plastic recycling in construction, then devotes considerable space to PET fibers in concrete and PET flakes in asphalt modification, even though the manuscript does not investigate either fibers or asphalt. It then addresses general, textbook-like topics such as conventional concrete, lightweight concrete, standards, and generic factors influencing strength, before returning, rather late, to the central topic: PET used as lightweight aggregate in lightweight concrete. This writing structure may confuse readers about the true scope of the study and weakens the relevance of the gap and the contribution of this research.

 I recommend a complete restructuring of the Introduction to maintain a coherent and concise narrative. In particular, the sections on PET fibers and asphalt should be removed or reduced to one or two sentences at most (for brief context purposes only), and the general information on concrete/lightweight concrete should be condensed to the bare minimum necessary to justify the performance targets and chosen classifications.

 At a minimum, the following writing structure is suggested:

- Motivation: Briefly present the challenge of PET waste and the relevance of its replacement as an aggregate in concrete. Here, please briefly include other applications (fibers, asphalt, etc.) of PET recycling in the construction materials/sector. Please include applications of recycled PET coatings, citing the following works: Resende D, et al., 2024 - Ref 1
  Alqahtani F, et al., 2017 - Ref 2
  Bórquez-Mendivil A, et al., 2026 - Ref 3
- State of the art: The use of PET as an aggregate substitute in concrete/lightweight concrete.
- Main technical barriers: For example, workability issues (hydrophobicity/angularity), challenges related to the interfacial transition zone/adhesion, mechanical tradeoffs, and concerns about durability/transport.
- Study focus and justification: Processing of PET with surface treatment, and why the experimental design and selected tests address these barriers.
- Gap and objectives: Clearly define what is missing and what this study contributes.

**Comment 2. Materials:** In Section 2.2 (Materials), calcium hypochlorite (Ca(ClO)₂) is introduced as part of the materials/methods description, but the manuscript does not clearly specify the intended purpose of using Ca(ClO)₂ in the context of this study. Please add one or two sentences stating why Ca(ClO)₂ was used (e.g., what it is intended to achieve for the PET aggregates) and briefly note any considerations associated with its use (e.g., how the authors ensured that its application would not introduce unintended effects relevant to cementitious performance).

- Section 2.6 refers to a previous publication by the same authors (Uche et al., 2024) for the thermal/mechanical production of PET aggregates, but the present manuscript does not provide a sufficiently self-contained description of the key processing steps and conditions. For reproducibility and interpretability, please include a concise summary of the workflow in this manuscript (e.g., shredding, melting/softening temperature range, cooling/solidification, crushing/sieving, targeted size fractions, and any basic quality-control checks). In addition, because the route involves thermal processing of PET, the “sustainable” framing should be bounded appropriately: please state the basic process boundary conditions (at minimum, whether ventilation/extraction was used) and explicitly acknowledge that energy demand and potential emissions were not quantified (no life cycle assessment /air-emissions assessment). This does not invalidate the materials results, but it is important to avoid over-claiming environmental benefit without measurement. A cross-reference to prior work is useful, but the critical processing parameters should still be briefly reported here to allow this article to stand independently.

**Comment 3. Experiment: **In Section 3.1, the manuscript states that the PET aggregates were chemically treated “to modify the surface of the aggregates to improve mechanical interlacing and chemical affinity for cement,” following a previously reported procedure (Lee et al., 2019). However, no surface-sensitive or interfacial characterization is provided to demonstrate that the treatment actually modified the PET surface (i.e., “modified surface” is asserted, but not evidenced). Figures 3a and 3b appear to show the aggregates visually, yet they do not verify chemical or physicochemical changes at the surface, nor do they establish how/why any change would translate into improved bonding.

 Please either, provide direct or at least minimal evidence supporting surface modification (e.g., a simple wettability indicator, comparative microscopy focused on surface texture/roughness, or an interface-related observation), or reframe the statement to avoid implying that surface modification was confirmed, describing the Ca(ClO)₂ step as a procedural pre-conditioning adopted from the literature and limiting mechanistic attribution accordingly.

 In addition, the text indicates that the treated aggregates were air-dried “to ensure that there was no residual solution on the surface,” but it remains unclear whether any rinsing/neutralization step was performed or how residue control was verified. Please clarify the post-treatment handling (washing/rinsing, drying conditions) and briefly discuss any considerations associated with introducing a chlorine-containing treatment in cementitious materials, so that the interpretation of mechanical/durability trends is not confounded by potential residual species.

- Overall, Sections 3.2–3.5 are more clearly reported than the Introduction and include useful standards/equations; however, two methodological points require clarification to ensure internal consistency and reproducibility.
- In Section 3.2, the selected w/c range (0.40–0.45) is justified as having proven ability to achieve a minimum compressive strength threshold (≥17 MPa) for structural lightweight concrete, yet the reported compressive strengths fall in the 14.1–16.5 MPa range. Please revise the wording in the experimental design rationale to align with the actual strength class achieved (e.g., explicitly framing the target classification used in the manuscript) and avoid using a threshold that is not met by the presented dataset.
- In Section 3.2.3 the mix design formulation includes an air-volume term ( *v _air_*) in the aggregate volume equations, but the manuscript does not explain whether *v _air_ *was measured, assumed, or fixed, nor how it was controlled across mixtures. Because PET aggregates can strongly influence entrapped air and hence density and strength, please clarify how air content was handled (measurement method or assumption) and indicate the value(s) used, so that the mix design and reported performance trends can be interpreted reproducibly.

**Comment 4. Results. **The Results section currently opens with a full summary table of all measured outputs and then repeats the interpretation in separate subsections. While a summary table is useful, placing it at the very start can reduce readability and may confuse the flow, as the reader is confronted with all outcomes before understanding the experimental logic and the stepwise interpretation. I recommend either (i) moving the global summary table toward the end of the Results section (or the end of the subsection sequence) as a consolidation tool, or (ii) retaining it early but significantly shortening it (or providing only the key response variables) and ensuring each subsection clearly points back to the relevant table entries. This would improve narrative clarity and reduce redundancy.

- Please correct the superscripts in the units shown in Figure 10 (e.g., proper formatting for m³, kg/m³, kPa·m³/kg, etc.). This is a minor formatting issue but affects technical presentation. Review all figures.
- Section 4.1 includes several mechanistic statements that are not supported by the reported macroscopic tests and are presented as definitive explanations. For example, after reporting differences between fresh and dry densities, the manuscript states: “This is because the voids in the concrete matrix were filled with the products of the cement hydration reactions.” This causal explanation cannot be concluded from density measurements alone without microstructural evidence (e.g., porosity measurements, microstructure/ITZ characterization, or hydration-product quantification), and it is currently presented without citation.
- Please reframe such statements as hypotheses or plausible interpretations (e.g., “may be associated with…”, “could reflect…”) and/or support them with appropriate references. Alternatively, if the authors wish to retain mechanistic explanations, they should provide direct supporting evidence (which does not appear to be included in the current manuscript).
- The discussion also states: “The concrete shows potential to be densified through proper proportioning of PET particle sizes and binder content, reducing common issues in PET concrete such as poor bonding and high porosity.” This statement introduces specific microstructural/ITZ claims (“poor bonding”, “high porosity”, “densification”) that are not directly demonstrated by the reported dataset (primarily fresh/dry density, strength, splitting tensile, absorption). If bonding/ITZ quality is a central argument, the manuscript would require corresponding interfacial or microstructural characterization, or the claim should be softened and carefully bounded.
- I recommend revising the language to avoid presenting “poor bonding” as a demonstrated or quantified issue in this study. At minimum, it should be framed as a known challenge reported in prior PET-aggregate literature (with citations) rather than a conclusion drawn from the current macroscopic results.

**Comment 5.** **Conclusions. **Improve the conclusions section by correlating the results obtained by each of the trials.

Is the work clearly and accurately presented and does it cite the current literature?

Partly

If applicable, is the statistical analysis and its interpretation appropriate?

Yes

Are all the source data underlying the results available to ensure full reproducibility?

Yes

Is the study design appropriate and is the work technically sound?

Yes

Are the conclusions drawn adequately supported by the results?

Partly

Are sufficient details of methods and analysis provided to allow replication by others?

Partly

Reviewer Expertise:

PET recycling, Sustainable Materials, Building Materials, Sol-gel, Polymers processing

I confirm that I have read this submission and believe that I have an appropriate level of expertise to confirm that it is of an acceptable scientific standard, however I have significant reservations, as outlined above.

## References

[1] : Sustainable Structural Lightweight Concrete with Recycled Polyethylene Terephthalate Waste Aggregate. *Buildings* .2024;14(3) : 10.3390/buildings14030609 10.3390/buildings14030609

[2] : Production of Recycled Plastic Aggregates and Its Utilization in Concrete. *Journal of Materials in Civil Engineering* .2017;29(4) : 10.1061/(ASCE)MT.1943-5533.0001765 10.1061/(ASCE)MT.1943-5533.0001765

[3] : Cement pastes modified with bis(2-hydroxypropyl) terephthalate derived from recycled polyethylene terephthalate: Chemical compatibility, microstructure, and fresh-hardened properties. *Construction and Building Materials* .2026;521: 10.1016/j.conbuildmat.2026.146105 10.1016/j.conbuildmat.2026.146105

# Reviewer report: Reviewer response for version 1

**DOI:** 10.5256/f1000research.185744.r424410

**Published:** 2025-11-28

**Authors:** Sandeep Sathe

**Affiliations:** 1 MIT World Peace University, Maharashtra, India

**Comment 1**. The title is clear but could be made more concise to highlight the core contribution (e.g., specify the material or performance parameter studied).

**Comment 2**. The abstract provides a good overview but should include quantitative results (e.g., percentage improvement or reduction in strength, durability, or cost).

**Comment 3**. The last sentence should emphasize the novelty and potential applications of the research instead of general statements.

**Comment 4**. Cite recent studies (2022–2025) from a reputable journal that discuss industrial waste reuse in SLWC. You may add the following research Article:

 1. Beibei X., et al., ( 2021) https://doi.org/10.1016/j.prostr.2021.10.114, 

 2. Sathe S, Rathod R (2025) https://doi.org/10.1007/s44290-025-00317-6,  

 3. Dumre G, et al., (2024) https://doi.org/10.1016/j.heliyon.2024.e39881, 

 4. Sathe S, Devsale A (2025) https://doi.org/10.1080/19648189.2025.2472055.

**Comment 5**. The introduction adequately covers background literature but needs a clear articulation of the research gap and how this work advances existing knowledge. And Clearly state the objectives and hypotheses at the end of the introduction to guide the reader.

**Comment 6**. Provide full chemical composition and physical properties of materials used (cement, supplementary materials, aggregates).

**Comment 7**. Mention the number of specimens tested per mix and include statistical treatment (average, standard deviation).

**Comment 8**. Clarify the mix proportion rationale—why specific replacement percentages were chosen.

**Comment 9**. Ensure testing standards (ASTM/IS/EN) are cited in full, not abbreviated.

**Comment 10**. Include a flow diagram summarizing the methodology to improve readability.

**Comment 11**. Discuss *why* certain trends occur (e.g., the relation between microstructure and mechanical strength).

**Comment 12**. Provide quantitative comparisons (e.g., “Compressive strength increased by 15% at 10% replacement compared to control”).

**Comment 13**. Use statistical or regression analysis if the data permit.

**Comment 14**. Enhance the discussion by correlating findings with previous studies.

**Comment 15**. Graphical representations (e.g., bar charts, stress–strain curves) can be improved for clarity and axis labelling consistency.

**Comment 16**. Discuss any anomalies or deviations in the data and possible explanations.

**Comment 17**. The conclusions are appropriate but should be rewritten in a concise, bullet-point format, emphasizing key findings and their practical implications.

**Comment 18**. Add a note on limitations (e.g., lack of long-term durability assessment or microstructural validation) and future work directions.

Is the work clearly and accurately presented and does it cite the current literature?

Yes

If applicable, is the statistical analysis and its interpretation appropriate?

Yes

Are all the source data underlying the results available to ensure full reproducibility?

Yes

Is the study design appropriate and is the work technically sound?

Yes

Are the conclusions drawn adequately supported by the results?

Yes

Are sufficient details of methods and analysis provided to allow replication by others?

Yes

Reviewer Expertise:

-

I confirm that I have read this submission and believe that I have an appropriate level of expertise to confirm that it is of an acceptable scientific standard.

## References

[1] : Experimental Characterization of Mortar with Recycled PET Aggregate: Preliminary Results. *Procedia Structural Integrity* .2021;33: 10.1016/j.prostr.2021.10.114 1027-1034 10.1016/j.prostr.2021.10.114

[2] : Experimental investigation of structural lightweight mortars containing waste PET aggregates. *Discover Civil Engineering* .2025;2(1) : 10.1007/s44290-025-00317-6 10.1007/s44290-025-00317-6

[3] : Investigation on properties of mortar with the use of simply shredded post-consumer plastic waste aggregate and molasses. *Heliyon* .2024;10(21) : 10.1016/j.heliyon.2024.e39881 10.1016/j.heliyon.2024.e39881 PMC1155012539524700

[4] : Influence of corrosion on bond strength in reinforced recycled aggregate concrete with fly ash. *European Journal of Environmental and Civil Engineering* .2025;29(10) : 10.1080/19648189.2025.2472055 1953-1986 10.1080/19648189.2025.2472055

# Reviewer report: Reviewer response for version 1

**DOI:** 10.5256/f1000research.185744.r421482

**Published:** 2025-11-06

**Authors:** Alejandro Meza-de Luna

**Affiliations:** 1 Tecnológico Nacional de México/IT Aguascalientes, Aguascalientes, Mexico

Congratulations on your paper and the work you have done. As with any research, there are areas for improvement to enhance indexing.

 The study lacks analysis and information to explain sustainable structural lightweight concrete, as indicated in the paper's title.

 In the introduction, please include additional applications of recycled PET fibers, such as: Meza, A. & Ahmed, F.U. (2020). Anisotropy and bond behaviour of recycled Polyethylene terephthalate (PET) fibre as concrete reinforcement. Construction and Building Materials, 265, 120331. Meza, A. & Siddique, S. (2019). Effect of aspect ratio and dosage on the flexural response of FRC with recycled fiber. Construction and Building Materials, 213, 286-291. Meza, A., Pujadas, P., Meza, L.M., Pardo-Bosch, F, & López-Carreño, R.D. (2021). Mechanical Optimization of Concrete with Recycled PET Fibres Based on a Statistical-Experimental Study. Materials, 14, 240.

 Check that all nomenclature is described at first time, i.e., in the Introduction section, indicate what SLWAC means.

 In the Materials section,  it is missing to indicate the characteristics of the cured specimens, such as temperature and humidity.

 Some Figures need improved visualization; i.e., enhance the quality of the labels into images, such as Figures 4, 7, etc.

 Check the units and format in all the documents, such as section 3.5, which says 0.25 NMM-2S-1, section 4.1 say 1488kg/m3 (check the separation).

 It is missing a specification of the test feed rate.

 It is better to explain the effect through changes in the parameters than to indicate it by a mix, i.e., mix 7. What difference did Mix 7 have with respect to others?.

 Section 3.3 does not specify if the DOE is complete or partial.

 Explain, Why do you not use repetition, like three or more elements, to analyze the variability?  

 Figure 12, Figure 14….. Check the labels; it's missing an indication of what 's.p..' means. F.A…..; also,  R2 is low. Is it pertinent to predict the equation?

 Pg 19, there are two introductions to Figure 14.

 The paer said, “As shown in Figure 14, the compressive strength of PET lightweight aggregate concrete exhibits a positive relationship with cement content and a negative relationship with water, fine aggregates, and coarse aggregate content”. How can you infer this if R² tends to zero?. Similar observations across all trends: R2 is almost zero.

 Explain why we do not use control specimens to make a comparison.

 It lacks an indication of the oven temperature for drying the specimens.

 It is necessary to conduct a study on the effects of parameter changes in the DOE, identifying which parameters influence the measured parameter.

 Check all the conclusion sections; i.e., conclusion number 6 is valid? Varius R ^2^ is near zero.

 It is missing conclusions about the parameter that you consider in DOE.

**Conclusion 3:** How does the article evidence that the mix 8 is an environmentally friendly option?

Is the work clearly and accurately presented and does it cite the current literature?

Partly

If applicable, is the statistical analysis and its interpretation appropriate?

Partly

Are all the source data underlying the results available to ensure full reproducibility?

Partly

Is the study design appropriate and is the work technically sound?

Yes

Are the conclusions drawn adequately supported by the results?

No

Are sufficient details of methods and analysis provided to allow replication by others?

Partly

Reviewer Expertise:

Materials, recycling

I confirm that I have read this submission and believe that I have an appropriate level of expertise to confirm that it is of an acceptable scientific standard, however I have significant reservations, as outlined above.

# Response

**Published:** 2025-12-31

**Authors:** Uche Chikadibia Kalu

**POINT-BY-POINT RESPONSE TO REVIEWERS**

**S/No.**

**Reviewer’s Comments**

**Author’s Response**

 1. In the introduction, please include additional applications of recycled PET fibers, such as: Meza, A. & Ahmed, F.U. (2020). Anisotropy and bond behaviour of recycled Polyethylene terephthalate (PET) fibre as concrete reinforcement. Construction and Building Materials, 265, 120331. Meza, A. & Siddique, S. (2019). Effect of aspect ratio and dosage on the flexural response of FRC with recycled fiber. Construction and Building Materials, 213, 286-291. Meza, A., Pujadas, P., Meza, L.M., Pardo-Bosch, F, & López-Carreño, R.D. (2021). Mechanical Optimization of Concrete with Recycled PET Fibres Based on a Statistical-Experimental Study. Materials, 14, 240.

**Response: **Additional applications of recycled PET fibers have been included. See lines 39 – 50.

 2. Check that all nomenclature is described at first time, i.e., in the Introduction section, indicate what SLWAC means.

**Response: **The nomenclature has been written out in full. See Line 125.

 3. In the Materials section,  it is missing to indicate the characteristics of the cured specimens, such as temperature and humidity.

**Response: **The characteristics of the curing location have been provided in Lines 325 – 326.

 4. Some Figures need improved visualization; i.e., enhance the quality of the labels into images, such as Figures 4, 7, etc.

**Response: **The quality of the labelling into the figures have been improved.

 5. Check the units and format in all the documents, such as section 3.5, which says 0.25 NMM-2S-1, section 4.1 say 1488kg/m3 (check the separation).

**Response: **The units and format have been improved in Lines 380 and 462.

 6.It is missing a specification of the test feed rate

**Response: **The feed rate for compressive strength test has been specified in Line 380

 7. It is better to explain the effect through changes in the parameters than to indicate it by a mix, i.e., mix 7. What difference did Mix 7 have with respect to others?.

**Response: **The difference between mix 7 and the others in terms of mixture proportions is contained in Table 6.

 8. Section 3.3 does not specify if the DOE is complete or partial.

**Response: **The complete replacement of natural aggregates has been specified in Lines 300 – 302.

 9. Explain, Why do you not use repetition, like three or more elements, to analyze the variability?

**Response: **The values provided in Table 7. The more detailed presentation of the result is contained in the supplementary files attached.

 10. Figure 12, Figure 14….. Check the labels; it's missing an indication of what 's.p..' means. F.A…..; also,  R2 is low. Is it pertinent to predict the equation?

**Response: **The indication of s.p and F.A. has been interpreted. The R ^2^ values are not predictive but rather indicative to support preliminary conclusions and provide a basis for optimization.

 11. Pg 19, there are two introductions to Figure 14.

**Response: **The dual introduction has been modified.

 12. The paer said, “As shown in Figure 14, the compressive strength of PET lightweight aggregate concrete exhibits a positive relationship with cement content and a negative relationship with water, fine aggregates, and coarse aggregate content”. How can you infer this if R² tends to zero?. Similar observations across all trends: R2 is almost zero.

**Response: **The R ^2^ values are not predictive but rather indicative to support preliminary conclusions and provide a basis for optimization.

 13. Explain why we do not use control specimens to make a comparison.

**Response: **Control specimens were not used for comparison because natural or artificial lightweight aggregates of similar physical and mechanical characteristics were not available in the location where this experiment was conducted.

 14. It lacks an indication of the oven temperature for drying the specimens.

**Response: **The temperature for drying the specimens has been indicated as 105 ^0^C. See Lines 412 – 413.

 15. It is necessary to conduct a study on the effects of parameter changes in the DOE, identifying which parameters influence the measured parameter.

**Response: **DOE based sensitivity analysis has been included in the recommendations for future studies in Lines 1046 – 1048.

 16. Check all the conclusion sections; i.e., conclusion number 6 is valid? Varius R ^2^ is near zero.

**Response: **The R ^2^ values are not predictive but rather indicative to support preliminary conclusions and provide a basis for optimization.

 17. How does the article evidence that the mix 8 is an environmentally friendly option?

**Response: **Mix 8 achieved a balance of sustainability and strength, by achieving acceptable strength with the highest content of PET aggregates, thus making it the most environmentally friendly option.
